# The Mechanistic Link Between Tau-Driven Proteotoxic Stress and Cellular Senescence in Alzheimer’s Disease

**DOI:** 10.3390/ijms252212335

**Published:** 2024-11-17

**Authors:** Karthikeyan Tangavelou, Kiran Bhaskar

**Affiliations:** 1 Department of Molecular Genetics and Microbiology, University of New Mexico Health Sciences Center, Albuquerque, NM 87131, USA; 2 Department of Neurology, University of New Mexico Health Sciences Center, Albuquerque, NM 87131, USA

**Keywords:** Alzheimer’s disease, tauopathy, tau, ubiquitin–proteasome system, autophagy, aggrephagy, nucleophagy, proteotoxic stress, cellular senescence, senolytic drugs

## Abstract

In Alzheimer’s disease (AD), tau dissociates from microtubules (MTs) due to hyperphosphorylation and misfolding. It is degraded by various mechanisms, including the 20S proteasome, chaperone-mediated autophagy (CMA), 26S proteasome, macroautophagy, and aggrephagy. Neurofibrillary tangles (NFTs) form upon the impairment of aggrephagy, and eventually, the ubiquitin chaperone valosin-containing protein (VCP) and heat shock 70 kDa protein (HSP70) are recruited to the sites of NFTs for the extraction of tau for the ubiquitin–proteasome system (UPS)-mediated degradation. However, the impairment of tau degradation in neurons allows tau to be secreted into the extracellular space. Secreted tau can be monomers, oligomers, and paired helical filaments (PHFs), which are seeding competent pathological tau that can be endocytosed/phagocytosed by healthy neurons, microglia, astrocytes, oligodendrocyte progenitor cells (OPCs), and oligodendrocytes, often causing proteotoxic stress and eventually triggers senescence. Senescent cells secrete various senescence-associated secretory phenotype (SASP) factors, which trigger cellular atrophy, causing decreased brain volume in human AD. However, the molecular mechanisms of proteotoxic stress and cellular senescence are not entirely understood and are an emerging area of research. Therefore, this comprehensive review summarizes pertinent studies that provided evidence for the sequential tau degradation, failure, and the mechanistic link between tau-driven proteotoxic stress and cellular senescence in AD.

## 1. Introduction

Tau is a natively unfolded and intrinsically disordered protein, which mainly associates with microtubules (MTs) to maintain the axonal integrity and synaptic plasticity by supporting the MT-based axonal trafficking of various cargoes back and forth from the cell body and the neuronal terminal [1]. In theory, intrinsically disordered proteins are subjected to different post-translational modifications, conformationally changed to gain novel functions, and interact with multiple proteins. Since tau is an intrinsically disordered protein, various kinases phosphorylate tau and alter its structure. This makes it acquire an unrecognized novel function depending on the context. The human *MAPT* gene encodes six isoforms of tau by alternative splicing. It is primarily expressed in neurons [1] and a small amount in mature oligodendrocytes (OLs) [2] in the central nervous system (CNS), and more than 30 different familial mutations in the *MAPT* gene cause frontotemporal dementia (FTD) and other tauopathy [3,4]. Intracellular accumulation of hyperphosphorylated tau as neurofibrillary tangles (NFTs) [5,6] is typical in tauopathy, including Alzheimer’s disease (AD). AD is the most prevalent tauopathy, where both amyloid beta (Aβ) [7,8] and NFT pathologies are evident [9,10,11]. Aging, metabolic dysfunction, oxidative stress, and neuroinflammation are risk factors that drive tau hyperphosphorylation, causing microtubule destabilization, neuronal instability, and eventually, neurodegeneration.

The pathological (hyperphosphorylated and/or misfolded) tau is a substrate for the proteasome and autophagy, as reviewed elsewhere [12,13,14]. However, the efficiency of these two degradative pathways declines during normal aging and tauopathy. Thus, it is essential to understand the mechanisms of pathological tau degradation and its impairment in various cell types in the brain, including neurons, microglia, astrocytes, and oligodendrocytes. Both newly synthesized and MT-dissociated tau undergo degradation, primarily in neurons. While the 20S proteasome can degrade unfolded tau, the 26S proteasome can degrade both misfolded and phosphorylated tau upon tagging with a K48-linked polyubiquitin chain and, thus, prevents tau from aggregating and seeding. However, the imbalance in protein homeostasis can exacerbate tau aggregation pathology because the cells cannot stop the protein translation upon impairment of the proteasomal activity and dysregulated molecular chaperones, which is often the case in various neurodegenerative diseases [15].

It is possible that the inability of the 26S proteasomal degradation of tau can lead to its hyperphosphorylation and differential ubiquitination, such as K63-linked polyubiquitin chains that can be a signal for either autophagy or exosomal secretion into the extracellular space. Notably, soluble tau oligomers are stabilized by K63-linked ubiquitin chains [16,17]. Therefore, tau undergoes hyperphosphorylation whenever the degradation pathway is impaired [18] and eventually gets ubiquitinated [19] for various protein degradation pathways [14,20,21]. To support this hypothesis, a high-resolution proteomics mapping of post-translational modifications (PTMs) in tau obtained from the autopsy of human AD brains revealed that tau can obtain 95 PTMs on 88 amino acid residues. They are mapped as 55 phosphorylation, 17 ubiquitination, 19 acetylation, and 4 methylation sites on tau. It suggests that PTMs can occur sequentially, leading to tau aggregation [18]. However, it is not clear whether tau needs to be hyperphosphorylated to dissociate from the MTs. Alternatively, sequential failure of tau degradation adds additional phosphorylation as a signal for differential linkage-specific ubiquitination in an attempt to push it through various degradative pathways. Protein homeostasis, including protein translation and degradation, is critical for cellular homeostasis, a regulated process that maintains cell growth and death. The impairment of protein homeostasis causes cells to become apoptosis-resistant senescent and secrete toxic contents and signaling molecules into the extracellular space through various mechanisms [14]. Given these complexities, this review article describes the dynamics of tau degradation and cellular senescence in AD.

## 2. Dynamics of Tau Degradation in AD

### 2.1. Ubiquitin-Independent Proteasomal Degradation of Tau in AD

**Figure 1:**
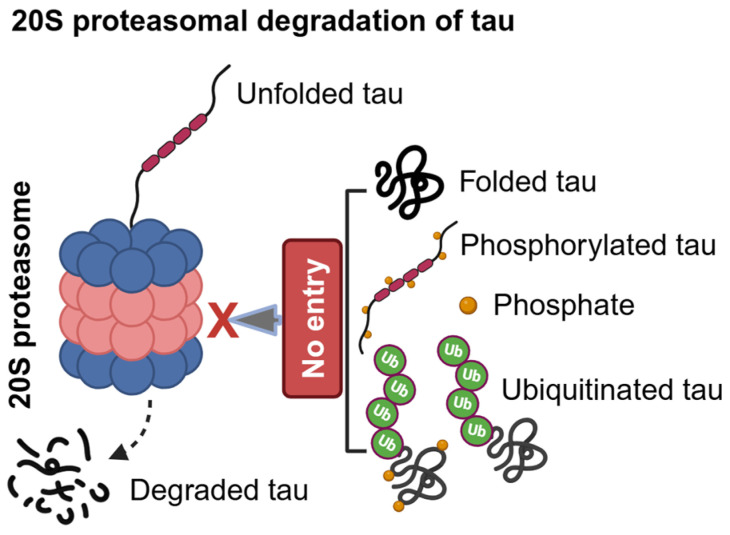
***20S proteasomal degradation of tau.*** The structure of the 20S proteasome consists of 7 different α subunit proteins (α1–α7—blue) and β subunit proteins (β1–β7—light red) in which β1, β2, and β5 subunits are constitutively catalytic active. The heptameric α subunits form an outer ring-like structure at the top and bottom of the inner two layers of heptameric β subunit proteins, stacked antiparallelly as an inner ring-like structure. The 20S proteasome can degrade natively unfolded tau. But, it does not degrade folded, phosphorylated (conformational changed), and ubiquitinated tau because of the absence of protein unfoldase and deubiquitinase activities, which are required to unfold and deubiquitinate tau before entering into the catalytic β subunit core proteins. Abbreviation: Ub, ubiquitin.

**Figure 2:**
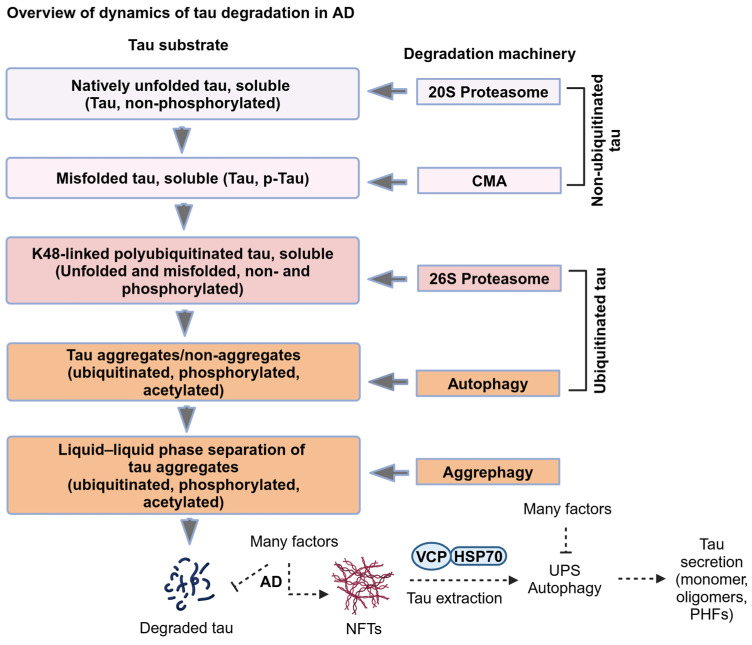
***Overview of dynamics of tau degradation in AD.*** The degradation pathway for tau is selected based on the diversity of tau species based on their structure and post-translational modifications. In AD, non-ubiquitinated tau substrates, including unfolded and misfolded tau, can be degraded by the 20S proteasome and CMA, respectively. Misfolded ubiquitinated tau substrates, including phosphorylated and acetylated tau, can be degraded by the 26S proteasome and macroautophagy (autophagy), respectively. Hyperubiquitinated aqueous tau aggregates are degraded by liquid-phase aggrephagy, whereas solid tau aggregates of NFTs with or without ubiquitin chains are degraded by solid-phase aggrephagy. The ubiquitin chaperone, VCP/p97 ATPase, binds to HSP70 and synergistically extracts tau from NFTs for the UPS or autophagy degradation. The pathological tau extracted from NFTs can be monomers, oligomers, and PHFs secreted into the extracellular space upon the impairment of the UPS or autophagy in neurons. Abbreviations: p-Tau, phosphorylated tau; CMA, chaperone-mediated autophagy; AD, Alzheimer’s disease; NFTs, neurofibrillary tangles; VCP, valosin-containing protein; HSP70, heat shock protein 70, UPS, ubiquitin–proteasome system; and PHFs, paired helical filaments.

Tau directly enters the catalytic core of the 20S proteasome, which is a ubiquitin- and ATP-independent proteolytic machinery that degrades mainly intrinsically disordered proteins. In vitro studies show that a recombinant human tau isoform (383 aa) can enter into the catalytic core of the 20S proteasome, purified from human erythrocytes and degrades tau, and with lactacystin, a specific 20S proteasome inhibitor stabilizes tau from undergoing degradation [22]. The brain’s longest human tau isoform (441 aa) has also been shown to be catalytically degraded by the 20S proteasome. Notably, calcium-calmodulin kinase II (CaMKII)-mediated tau phosphorylation at S262, S324, S352, and S356 has been shown to abolish the 20S proteasomal activity completely. In contrast, glycogen-synthase kinase-3 beta (GSK3β)-mediated tau phosphorylation at S46, T175, T181, S202, T205, T212, T217, T231, S235, S396, S400, and S404 inhibited 20S proteasomal activity partially [23]. This study suggests that the 20S proteasome may not degrade phosphorylated tau, which is perhaps misfolded and cannot be traversed effectively into the 20S proteasome for degradation (Figure 1 and Figure 2).

Moreover, the intrahippocampal injection of lactacystin, which is a 20S proteasome inhibitor, led to the accumulation of insoluble ubiquitinated tau in rat brains, which surprisingly was not hyperphosphorylated despite increased cAMP-dependent protein kinase A (PKA), GSK-3β, and cyclin-dependent kinase-5 (Cdk-5) activities, and decreased protein phosphatase-2A (PP2A) activity [24]. However, the increased accumulation of ubiquitinated tau by lactacystin did not affect learning and memory [24]. It suggests that acute 20S proteasomal inhibition does not increase tau phosphorylation and preferentially degrades unfolded and non-phosphorylated tau. Lactacystin-mediated accumulation of insoluble ubiquitinated tau indicates that 20S proteasome may also degrade ubiquitin-conjugated unfolded soluble tau. Alternatively, tau is ubiquitinated upon the inhibition of 20S proteasome for either 26S proteasomal or autophagic degradation. To support the first speculation, the 20S proteasome has been shown to degrade K48-linked mono to tetra ubiquitin conjugated intrinsically disordered cyclin B1 peptide (1–88 aa) wherein ubiquitin conjugate also degraded, and 20S’s mechanism of action is distinct from 26S proteasome, which recycles ubiquitin from the substrates via deubiquitination [25]. Thus, it is suggested that the 20S proteasome plays a pivotal role in regulating tau levels in neurons, and inhibiting its activity is sufficient to cause total tau accumulation and disease progression in an early stage before tau hyperphosphorylation and tau seeding into NFTs.

### 2.2. Molecular Chaperone-Assisted Ubiquitin-Dependent Proteasomal Degradation of Tau in AD

The 26S proteasome recognizes substrates through the 19S regulatory proteasome submit complex by interacting with polyubiquitin chains followed by the sequential action of unfolding and deubiquitinating the substrates before entering the main catalytic 20S proteasome submit complex for degradation. As mentioned above, tau is a natively unfolded protein bound to the microtubules. In AD, phosphorylated tau is detached from MTs and undergoes aggregation driven by three pro-aggregation motifs mapped at R2 (275–280 aa), R3 (306–311 aa), and R4 (337–342 aa) in the microtubule-binding repeats [1,26]. Upon conformational changes in tau, the molecular chaperone is recruited to prevent aggregation and restore its native state or direct it to the ubiquitin–proteasome system (UPS) for degradation [15].

Phosphorylated tau can bind to the molecular chaperones heat shock protein 70 (HSP70) or heat shock cognate 71 kDa protein (Hsc70), which in turn recruits the E3-ubiquitin ligase C-terminus of Hsc70-interacting protein (CHIP) and E2-ubiquitin-conjugating enzyme UbcH5B to attach K48-linked polyubiquitin chains on tau covalently for the 26S proteasomal degradation [27,28]. The molecular cochaperone BCL2 Associated Athanogene 1 (BAG1) functions as an ATP/ADP exchange factor of HSP70 or Hsc70 to release the properly refolded proteins. Also, it enhances the anti-apoptotic effects of B-cell lymphoma 2 (BCL2) [29,30]. The overexpression of BAG-1 in neurons inhibited the proteasomal activity of the 20S but not the 26S, leading to an increased accumulation of total tau and its ubiquitination level [31]. Thus, it is suggested that BAG-1 favors tau refolding via Hsc70 and ubiquitination for the 26S proteasomal degradation. To support this hypothesis, BAG-1 depletion decreased the total tau accumulation, perhaps the consequence of enhanced 20S proteasomal activity, and inhibited tau refolding by reducing the interactions of Hsc70 [31]. The remaining undegraded tau from BAG-1 depleted cells undergo phosphorylation. Furthermore, endogenous BAG-1 is associated with hyperphosphorylated tau aggregates in 3xTg AD transgenic mice [31].

**Figure 3:**
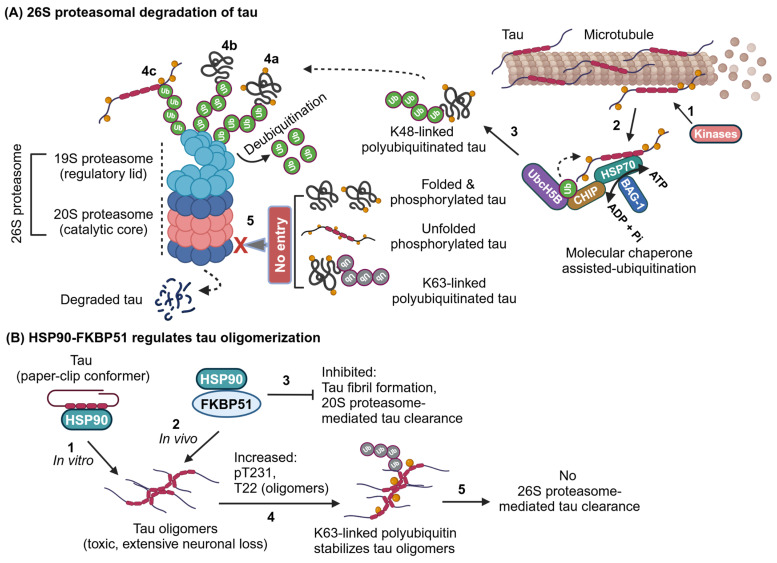
***Molecular chaperones regulate tau degradation and stability via ubiquitination.*** (**A**) The 26S proteasomal degradation of tau. In AD, MT-bound tau can be phosphorylated by various kinases such as PKA, CaMKII, GSK-3β, and Cdk-5, causing tau to detach from MT (1). Then, aggregation-prone phosphorylated tau is recognized by HSP70/Hsc70 and its cochaperone BAG-1 to assist K48-linked polyubiquitination by the E3-ubiquitin ligase CHIP and E2-ubiquitin-conjugating UbcH5B protein complex (2). BAG-1 functions as a nucleotide exchange factor to release the misfolded and ubiquitinated tau from chaperones (3). The 26S proteasome consists of the inner catalytic core 20S proteasome and the outer regulatory 19S proteasome subunits, which recognize the ubiquitinated tau through binding to its ubiquitin chains and immediately unfold and deubiquitinate before entering into the 20S proteasome for tau degradation. The ubiquitinated tau substrate can be (4a) folded, (4b) phosphorylated and folded, and (4c) phosphorylated and unfolded. The non-ubiquitinated tau, including phosphorylated and non-phosphorylated folded tau, unfolded phosphorylated tau, and K63-linked polyubiquitinated tau, are not substrates for the 26S proteasomal degradation (5). (**B**) HSP90-FKBP51 regulates tau oligomerization. During in vitro conditions, HSP90 binds to paper-clip conformers of tau and promotes their oligomerization, which involves MTBRs (1). During in vivo conditions, HSP90 binds to its co-chaperone FKBP51 and enhances tau oligomerization (2), but this interaction does not allow transition into Thioflavin-T positive fibrillar tau (3). The HSP90-FKBP51 complex synergistically inhibits 20S proteasome-mediated degradation of tau (3), leading to the accumulation of phosphorylated tau (pT231) and toxic tau oligomers (T22 positive oligomers) (4). Tau oligomers can be stabilized by K63-linked polyubiquitin chains, which cannot be degraded by the 26S proteasome (5) unless it obtains K48-linked ubiquitin as a hybrid K63/K48-linked ubiquitination. Abbreviations: Ub, ubiquitin; CHIP, C-terminus of Hsc70-interacting protein; HSP70, heat shock protein 70; BAG-1, BCL2 Associated Athanogene-1; UbcH5B, ubiquitin-conjugating enzyme E2D 2; ATP, adenosine triphosphate; ADP, adenosine diphosphate; Pi, inorganic phosphate; HSP90, heat shock protein 90 kDa; FKBP51, FK506 binding protein 51 kDa; pT231, phosphorylated Tau at threonine 231; T22, tau oligomer specific antibody; and MTBRs, microtubule-binding repeats.

To conclude this section, the Tau-Hsc70-BAG-1 complex favors tau refolding and inhibits the 20S proteasomal activity, and eventually, Hsc70 recruits the CHIP-UbcH5B complex for K48-linked polyubiquitination for the 26S proteasomal degradation. Thus, BAG-1 likely serves as a transition factor that switches tau from being a 20S proteasomal substrate into a 26S proteasomal substrate. It is possible that the drugs targeting to accelerate the 20S proteasomal activity by inhibiting the BAG-1 nucleotide exchange factor could be an effective therapeutic strategy for tauopathy at the early stage before NFT formation in neurons (Figure 3A).

The other family of heat shock protein Hsp90 binds to the paper-clip conformer of tau to predispose the pro-aggregation motifs in the microtubule-binding repeat domains, causing tau to oligomerize differentially. However, these paper-clip-derived tau oligomers inhibited fibril formation and favored the continuation of tau oligomerization [32]. However, when heat shock protein 90 kDa (HSP90) interacts with FK506 binding protein 51 kDa (FKBP51), a cochaperone inhibits the 20S proteasomal activity and favors tau to oligomerize, which exacerbates neurotoxicity in rTg(tauP301L)4510 mice [33] (Figure 3B). Hsp90 can also bind to CHIP and selectively clears phosphorylated tau [34]. Notably, the deletion of CHIP in JNPL3 (P301L) mice enhanced the accumulation of the Sarkosyl-soluble higher molecular weight of non-aggregated tau, which is hyperphosphorylated and non-ubiquitinated. This study suggests that CHIP-mediated polyubiquitination may play a pivotal role in the formation of Sarkosyl-insoluble tau aggregates formation in disease conditions [35]. Phosphorylated tau oligomers can be stabilized by K63-linked ubiquitin, which makes them resistant to 26S proteasome-mediated degradation (Figure 3B). Interestingly, “tau oligomers purified from the autopsy of human AD brains” cause secretion of tau from iHEK-tau reporter cells overexpressing K63-linked ubiquitin augments seeding activity in tau biosensor cells. However, this is not the case in K48-linked ubiquitin-expressing reporter cells, which implies that tau oligomers carrying K63-linked ubiquitin chains propagate cell-to-cell tau seeding [16,17]. Similar to BAG-1, HSP90 co-chaperone FKBP51 inhibits the 20S proteasomal activity. CHIP is the main E3-ligase for tau ubiquitination and can function with either Hsc70 or HSP90 in pathological conditions. Finally, K63-linked ubiquitin stabilizes tau oligomers and promotes more tau propagation.

### 2.3. LC3-Independent Chaperone-Mediated Autophagy (CMA) Degradation of Tau in AD

To overcome failed proteasomal degradation of tau, neurons engage in lysosome-mediated degradation by LC3-independent chaperone-mediated autophagy (CMA) and LC3-dependent macroautophagy. LC3 is a ubiquitin-like molecule known as microtubule-associated protein 1 light chain 3 (MAP1LC3) or autophagy-related protein 8 (ATG8), and its lipidated form LC3-PE level is used as a marker for autophagy flux. The CMA-associated molecular chaperone Hsc70 recognizes the KFERQ motif-containing folded or unfolded soluble proteins and facilitates its interaction with lysosome-associated membrane protein-2A (LAMP-2A, also known as CD107b or Mac-3), which oligomerizes to form a channel-like structure at the lysosomal membrane to deliver the substrates for lysosomal degradation [36,37,38]. Inactivating CMA by specifically deleting LAMP-2A in mouse excitatory neurons causes proteotoxic stress and accumulation of CMA, but not LC3-dependent autophagy, substrates, and exacerbation of pathology in the 3xTg-AD mouse model [39]. In the LAMP-2A knockout neurons, autophagy does not compensate to degrade the CMA substrates (38), but it does in non-neuronal proliferating cells and mouse fibroblasts (NIH 3T3) [40]. Thus, it is suggested that neuronal autophagy is unique among other cell types in the brain. Furthermore, CMA activity is essential in synaptic remodeling, as it selectively eliminates synaptic proteins containing the KFERQ motif [39].

**Figure 4:**
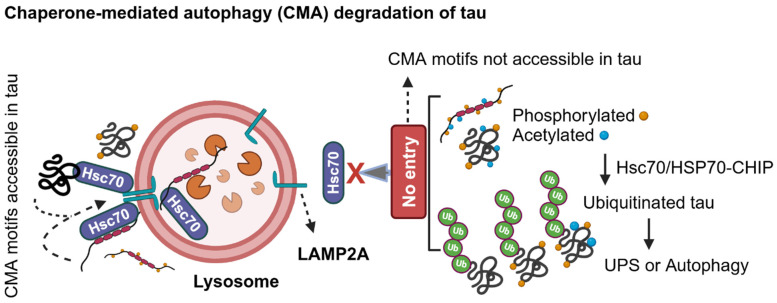
***Chaperone-mediated autophagy (CMA) degradation of tau*.** The molecular chaperone Hsc70 binds to CMA motifs in tau and eventually interacts with the lysosome-associated membrane protein 2A (LAMP2A), which oligomerizes to form a channel-like structure to deliver tau along with Hsc70 into the lumen of the lysosome for degradation. Hsc70 stays with tau until the proteases digest tau. If not, tau gets aggregated in an acidic environment, leading to the generation of amyloidogenic tau fragments. The CMA can degrade tau if the CMA motifs can be easily accessible in either folded or unfolded tau. Acetylated tau is not a substrate of CMA, and it can eventually obtain ubiquitin chains for either autophagy or 26S proteasomal degradation. Abbreviations: HSP70, heat shock protein 70; Hsc70, heat shock cognate 71 kDa protein; CHIP, C-terminus of Hsc70-interacting protein; UPS, ubiquitin–proteasome system; CMA, chaperone-mediated autophagy; LAMP2A, lysosome-associated membrane protein 2A; and Ub, ubiquitin.

Tau consists of two CMA-like motifs at ^336^QVEVK^340^ and ^347^KDRVQ^351^ in 2N4R isoform–both reside at the microtubule-binding repeating domain R4, which is present in all six isoforms of tau. Mutating these two CMA motifs in tau attenuated interaction with Hsc70, causing inefficient tau cleavage, leaving a seed-competent tau fragment that has a tendency to aggregate [39]. The CMA motifs in tau can also be perturbed by post-translational modifications or frontotemporal dementia and Parkinsonism linked to the chromosome 17 (FTDP-17)-associated tau ΔK280 deletion mutation that affects tau mRNA splicing [41]. Tau is preferably degraded by the 20S proteasome, which can degrade unfolded proteins. In pathological conditions, Hsc70 can recognize phosphorylated tau to prevent aggregation and interact with either CHIP for UPS- or LAMP2A for CMA-mediated degradation (Figure 4). Notably, Hsc70 can also shuttle the unmodified tau monomer, acetylated tau, and tau oligomers into the late endosomes for microautophagy-mediated degradation or multivesicular body-associated exosomes containing tau secretion into the extracellular space [42].

### 2.4. LC3-Dependent Autophagy Degradation of Tau in AD

**Figure 5:**
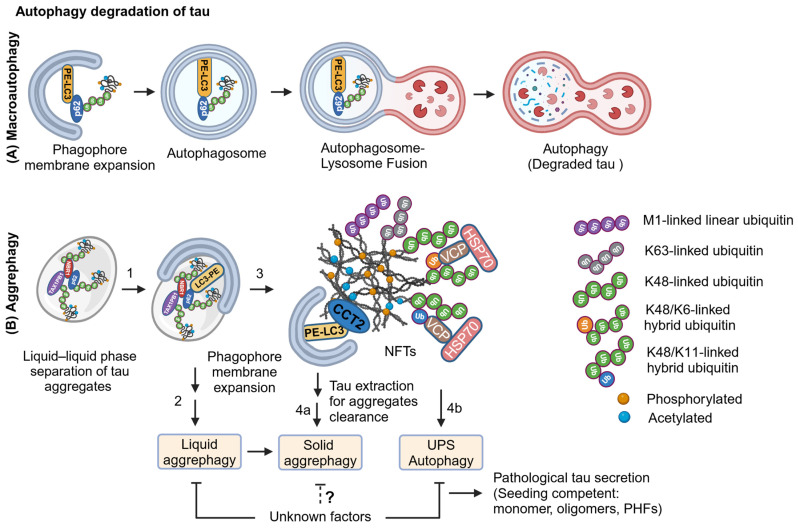
***Autophagy degradation of tau*.** (**A**) Macroautophagy (autophagy) can degrade ubiquitinated non-aggregates or aggregates of tau upon the impairment of the ubiquitin–proteasome system (UPS). An autophagy receptor p62 (SQSTM1) recognizes polyubiquitinated tau as an autophagy cargo and interacts with LC3 (ATG8), which is lipidated with phosphatidylethanolamine (PE) to anchor cargo at the inner leaflet of the lipid bilayer for phagophore membrane expansion. Autophagosomes formed against tau can fuse to lysosomes for tau degradation, which is known as macroautophagy (autophagy). (**B**) Aggrephagy degradation of tau aggregates. Hyperubiquitinated tau aggregates within the aqueous phase are recognized by the p62 receptor initially and subsequently recruit other SQSTM1-like receptors (SLRs), including the next to BRCA1 gene 1 protein (NBR1) and Tax1 binding protein 1 (TAX1PB1) via their ubiquitin-binding domain (UBA). Tau aggregates are condensed via liquid–liquid phase separation (LLPS) (1) and recruit autophagy regulatory proteins, including LC3, for phagophore membrane expansion for liquid aggrephagy-mediated clearance of tau aggregates (2). However, some unknown factor(s) impair autophagosome formation or autophagosome maturation with the lysosome, leading to the formation of membrane-less solid aggregates of NFTs, which can be differentially marked with various linkage-specific ubiquitin chains (3). The molecular chaperone aggrephagy receptor chaperonin containing TCP1 subunit 2 (CCT2) can recognize solid protein aggregates via its apical domain for solid aggrephagy-mediated clearance of tau aggregates with or without ubiquitin chains (4a). The ubiquitin chaperone valosin-containing protein (VCP)/p97 ATPase extract ubiquitinated tau aggregates (K48/K6, K48/K11, or K48/K63 hybrid ubiquitin chains) for either autophagy or UPS-mediated degradation (4b). However, pathological tau, including monomers, oligomers (K63-linked or K48/K63-linked hybrid ubiquitin chains), and PHFs, are secreted into the extracellular space upon the impairment of autophagy or UPS. NFTs are marked with M1-linked linear ubiquitin chains for NF-κB associated inflammatory signaling activation in AD brains. Abbreviations: Ub, ubiquitin; LC3-PE, phosphatidylethanolamine conjugated to C-terminus of microtubule-associated protein 1 light chain 3; UPS, ubiquitin–proteasome system; HSP70, heat shock protein 70; and NFTs, neurofibrillary tangles.

The CMA activity is inhibited upon tau acetylation, thus promoting tau as a substrate for macroautophagy (autophagy) [42], which can degrade small aggregates to large tau-paired helical filaments by encapsulating them in a double membranous autophagosome and eventually fuse with lysosome for degradation (Figure 5A). The zinc-dependent histone acetyltransferase p300/CBP acetylates, and NAD^+^-dependent sirtuin 1 (SIRT1) deacetylates tau [43]. SIRT1 level is decreased in aging, AD, and other neurodegenerative diseases [42,43], and tau is hyperacetylated in the absence of SIRT1, causing acetylated (and also phosphorylated) tau to accumulate [43]. Tau acetylation at K280 [44], localized in a pro-aggregation motif in the R2 microtubule-binding domain, destabilizes microtubules and forms tau fibrillization. Moreover, K280 acetylated tau is mainly present in Thioflavin-S positive insoluble protein inclusions in autopsy human AD brains and transgenic AD animal models, including PS19 (Tau P301S) and PS19; PDAPP (PS19 mice expressing APP V717F) [44]. The link between tau acetylation and hyperphosphorylation has been shown in the human neuroblastoma cell line SH-SY5Y overexpressing acetyltransferase p300, which acetylates tau at K280 causing its phosphorylation on S199, AT8 (S202, T205), AT180 (T231), S262, and S422 [45] and accumulation. The combinations of 2N4R tau phosphorylation by glycogen synthase kinase (GSK)3β and ubiquitylation with CHIP in the presence of acetyltransferase p300 facilitate tau degradation by 26S proteasome. However, prolonged ubiquitination on tau leads to the accumulation of hyperubiquitinated insoluble tau fibrils [46], which correlates with overall increased ubiquitylome and inefficient 26S function in autopsy human AD brains [46]. It is possible that upon impairments of the UPS and CMA, tau can be ubiquitinated with K63-linked polyubiquitin chains for autophagy degradation. p62 can recognize autophagy cargo by interacting with K48- or K63-linked polyubiquitin chains and recruit ubiquitin-like protein LC3 conjugated with phosphatidyl ethanolamine (LC3-PE) for autophagosome formation. Thus, autophagosomes formed around tau can fuse with lysosomes for autophagy, which can degrade soluble and aggregated tau (Figure 5A). However, the mechanism of autophagy degradation of tau is unknown.

### 2.5. Decoding of Hyperubiquitinated Tau Fibrils

The tandem mass spectrometry analysis (MS/MS) of ubiquitinated tau PHFs isolated from autopsied human AD brains suggests that PHFs are subjected to various polyubiquitination, including M1-, K6-, K11-, K48-, and K63-linked polyubiquitination [47,48,49]. Ubiquitin is a signaling protein consisting of 76 amino acid residues and is ubiquitously expressed in eukaryotic cells. Ubiquitination is a complex post-translational modification on target proteins that can be stabilized or degraded depending on the ubiquitin-linkage specificity. The terminal amino acid of ubiquitin is glycine, which can be covalently attached to the target proteins at methionine (M), lysine (K), cysteine (C), serine (S), and threonine (T). The subsequent ubiquitination occurs on the first ubiquitin attached to the protein substrates via ubiquitin internal residues at M1, K6, K11, K27, K29, K33, K48, and K63. Protein substrates can be monoubiquitinated or multi-monoubiquitinated and polyubiquitinated with homotypic or heterotypic chains that can be linear or branched ubiquitin chains [50]. The K48-linked polyubiquitination on tau is a signal for 26S proteasomal degradation, whereas K63-linked polyubiquitination eventually occurs on the aggregated tau already carrying K48-linked polyubiquitin chains upon the impairment of proteasomal activity (Figure 5B). The linear ubiquitin chain assembly complex (LUBAC), which includes the Ring finger protein 31 (RNF31)-Heme-oxidized IRP2 ubiquitin ligase 1 (HOIL1)-SHANK associated RH domain interactor (SHARPIN) complex, an M1-specific E3-ubiquitin ligase, can generate M1-linked linear polyubiquitin chains on tau fibrils to mark tau as an inflammatory molecule (Figure 5B), and eventually recruit the inhibitor of κB (IκB) kinase complex (IKKα-IKKβ-IKKγ) by interacting with the NF-κB essential modulator NEMO (IKKγ) for NF-κB downstream signaling activation. The LUBAC-based M1-linked polyubiquitination has been identified in various proteinopathies, including polyglutamine diseases, amyotrophic lateral sclerosis, frontotemporal dementia, and AD [51,52,53]. The NEMO can bind and ubiquitinate with M1-linked linear polyubiquitin chains by LUBAC upon stimulation with interleukin-1β (IL-1β) and NEMO phase separation, essential for IL-1β induced NF-κB activation [54].

IL-1β and tumor necrosis factor α (TNFα) have been shown to induce K63-linked polyubiquitination on TNF receptor-associated factor 6, E3 ubiquitin ligase (TRAF6) by itself, receptor-interacting protein (RIP1) by TNF receptor-associated factor 2, and E3 ubiquitin ligase (TRAF2), respectively. The K63-linked polyubiquitin chains are recognized by scaffolding proteins TAK1 binding protein 2 (TAB2) and the IKKα-IKKβ-IKKγ complex via NEMO (IKKγ). TGFβ-activated kinase 1(TAK1) binds to TAB2 and phosphorylates IKKβ, which in turn phosphorylates the NF-κB inhibitor IκBα for downstream activation of NF-κB signaling [55,56]. Interestingly, TAX1PB1 binds to deubiquitinase TNFα -induced protein 3 (TNFAIP3 or A20) and negatively regulates IL-1β and TNFα-induced NF-κB activity by deubiquitinating TRAF6 and RIP1 [57]. Thus, it suggests that TAX1BP1 likely plays a role in the crosstalk between aggrephagy and inflammation.

The ubiquitin chaperone VCP/p97 ATPase has been shown to extract the formaldehyde-induced cross-linked proteins from RNA by recognizing the K6-linked ubiquitin chains on a subset of proteins for the 26S proteasomal degradation [58,59] (Figure 5B). The 26S proteasome mainly degrades the K11-linked or hybrid K11/K48-linked polyubiquitinated proteins [60]. VCP can also bind and extract the hybrid K11/K48-linked polyubiquitin chains for rapid proteasomal degradation of newly synthesized misfolded and aggregation-prone proteins [50,61,62] (Figure 5B). However, the significance of unconventional K6- or K11-linked polyubiquitination on tau fibrils is unknown.

### 2.6. Aggrephagy Degradation of Tau Aggregates

Aggrephagy is a selective autophagy degradation of protein aggregates by encapsulating them in a double membranous autophagosome and subsequently fusing with a lysosome for clearance of protein aggregates [63]. To some extent, hyperubiquitinated and seed-competent/aggregated tau can be selectively degraded by aggrephagy (Figure 5B). Protein aggregates in the aqueous phase can be recognized by aggrephagy receptors, including p62 (SQSTM1/Sequestosome 1), the Next to BRCA1 gene 1 protein (NBR1), the Tax1 binding protein 1 (TAX1PB1), Optineurin (OPTN), and the Toll-interacting protein (TOLLIP). These receptors have an LC3-interacting region motif (LIR) and ubiquitin-binding domain (UBA) to interact with the autophagy protein LC3 and ubiquitinated protein aggregates cargo, respectively, for aggrephagy-mediated protein aggregates clearance [63,64,65]. A solid protein aggregate can be recognized by another aggrephagy receptor chaperonin containing T-complex polypeptide 1, subunit 2 (CCT2), which is a component of the chaperonin T-complex protein ring complex (TRiC). CCT2 has two LIR-like motifs (VLL and VIL amino acid residues) known as V-LIR that interact with LC3, but it does not have a UBA domain; instead, it interacts with protein aggregates cargo via its chaperoning apical domain [66,67]. Notably, CCT2 can interact with ubiquitinated or non-ubiquitinated protein aggregates cargo via its apical domain for solid protein aggregates clearance [66].

Autophagy receptor p62 is recruited that preferentially interacts with K63- over K48-linked polyubiquitin chains conjugated to tau aggregates for p62–ubiquitin mediated segregation of protein aggregates as larger condensates through liquid–liquid phase separation (LLPS) [49,67,68,69]. Then, the sequential action of recruiting other SQSTM1-like receptors (SLRs), including NBR1 and TAX1PB1, but not the Nuclear domain 10 protein 52 (NDP52) and OPTN, is indispensable for the efficient clearance of protein aggregates by aggrephagy [70,71]. Tau aggregates condensation via LLPS is similar to amyotrophic lateral sclerosis (ALS)-associated protein aggregates of Fused in Sarcoma (FUS), hnRNPA1, and TAR DNA-binding protein 43 (TDP43) [72]. Tau fibrils extracted from the human AD brain do not interact with the aggrephagy receptor TAX1PB1 because its binding site is masked in hyperubiquitinated tau [71]. TAX1PB1 deficiency causes the accumulation of both ubiquitinated proteins and premature aging marker lipofuscin in mouse brain tissues [73]. Thus, it suggests that a missing or masking of a unique linkage-specific ubiquitin chain impairs TAX1PB1 to effectively promote aggrephagy clearance of protein aggregates, eventually building NFTs in neurons (Figure 5B).

### 2.7. Tau Extraction from Hyperubiquitinated Neurofibrillary Tangles

In the tau propagation model, VCP and HSP70 cooperatively promote the disaggregation of polyubiquitinated tau fibrils for the 26S proteasomal degradation [74] (Figure 5B). However, the impaired degradation of disaggregated tau can become seeding competent in the recipient cells [74]. Moreover, VCP/p97 ATPase expression is decreased in the frontal cortex of human AD brains [75]. Overexpressing VCP in zebrafish embryos and mice expressing human Tau P301L induces autophagy-mediated clearance of tau aggregates and consequently decreases phosphorylated tau (AT8, S199, and S396) in NFTs [75]. It is possible that K6-, K11-linked, or a hybrid with K48-linked ubiquitination on tau fibrils may allow VCP to extract tau from NFTs for either the UPS or autophagy-mediated degradation (Figure 5B). Tau can also be secreted into the extracellular space upon the impairment of the UPS and autophagy in AD. Because of the technical limitations, the sequential action of different types of unconventional ubiquitin-specific linkages, such as K6- and K11-linked polyubiquitin, and their hybrid ubiquitination with K48 or K63 on tau fibrils has not been explored well to better understand the temporal consequences of these on tau degradation in AD and tauopathy.

The dynamics of tau structure, including natively unfolded to pathologically misfolded monomers, oligomers, PHFs, and NFTs, and various post-translational modifications such as acetylation, phosphorylation, and ubiquitination determine the appropriate degradative pathway or exploit the degradative pathway as the tau seeding platform for cell-to-cell pathological tau propagation (Figure 5B and Figure 2). Despite this, there is evidence for tangle-bearing neurons in the brains of AD [76,77]. If so, how do tangle-bearing neurons survive upon proteotoxic stress driven by the impairment of the UPS and autophagy? Moreover, the post-mitotic neurons cannot dilute the toxic misfolded protein aggregates through cell division, which often occurs in proliferating cells to prevent proteotoxic stress [78]. In order to survive, neurons containing pathological tau can either undergo cell-cycle activation and get arrested at a specific cell-cycle stage [79] or trigger proteotoxic stress-associated senescence [80,81].

## 3. Cellular Senescence in Alzheimer’s Disease (AD)

### 3.1. Cellular Senescence

The term cellular senescence, in which the cell cycle is permanently arrested, differs from cellular quiescence, where the cell cycle is temporarily paused at the G0 phase to arrest the cell division/proliferation [82]. Neurons are terminally differentiated and non-proliferative cells indefinitely attain a quiescent phase. How neurons acquire a senescence-like phenotype in the absence of the cell cycle in normal human aging [83,84] or neurodegenerative disease brains [85] is unclear. Senescent cells are pro-inflammatory and have senescence-associated secretory phenotype (SASP) characteristics, including the secretion of cytokines, chemokines, growth factors, and metalloproteases [86]. On the other hand, quiescent cells are not pro-inflammatory and can re-enter the cell cycle. However, there is an overlap between the cellular senescence and quiescence. Identifying biomarkers to distinguish these two cellular phenotypes is an active area of research [87]. Therefore, understanding the molecular mechanisms driving the onset and expansion of neuronal senescence is paramount to identifying novel biomarkers and developing effective therapeutic strategies against pro-inflammatory senescent cells.

In addition to proteostatic stress, cellular senescence can also be induced by other intrinsic factors such as the shortening of telomeres, oxidative stress, reactive metabolites, DNA damage repair defects, epigenetic changes, oncogene activation, and some external factors, including chemotherapeutics, radiation and UV light exposure, mutagenic molecules, and viral infections [88]. Many biomarkers are available to detect the senescence cells, including the cell cycle-independent accumulation of lipofuscin and senescence-associated β-galactosidase (SA-β-gal) in the lysosomes and decreased level of nuclear membrane protein lamin B. The increased level of cyclin-dependent kinase inhibitors, which arrest the cell cycle at the G1 phase such as p16 (CDKN2A/INK4), p19 (CDKN2D/INK4D), p21 (CDKN1A), and phospho-p53 (tumor suppressor p53), phospho-retinoblastoma protein (pRB), and absence of cell proliferation marker Ki-67 are biomarkers of cellular senescence [86,88,89]. SASP contents vary among the cell types and factors that induce primary senescence, whose SASP contents induce secondary senescence by paracrine and juxtacrine or a combination of both to other healthy cells (astrocytes, microglia, oligodendrocytes, and other non-neuronal cells) in the brain [90].

### 3.2. Senescence in Neurons

**Figure 6:**
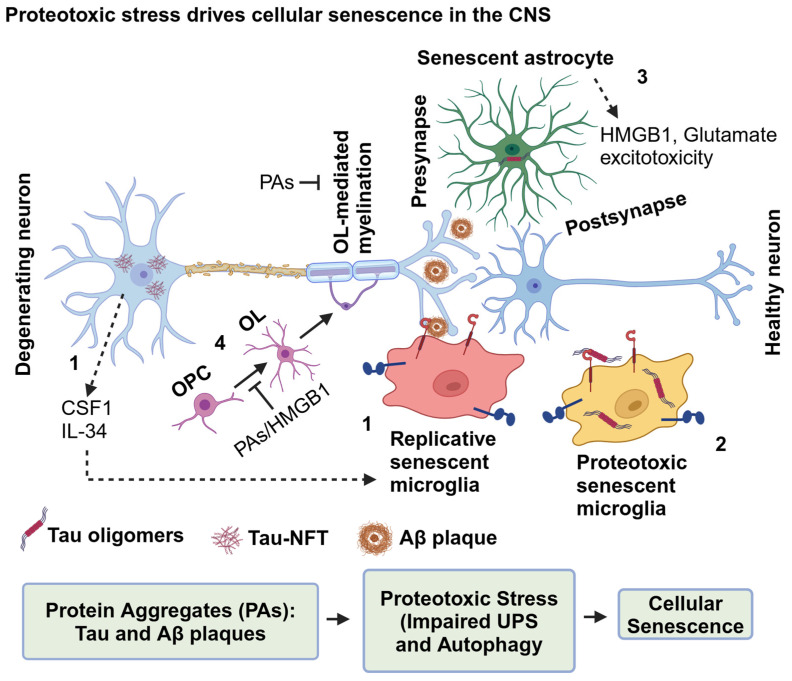
***Proteotoxic stress drives cellular senescence in AD*.** Pathological tau and Aβ drive proteotoxic stress in neurons by impairing the UPS and autophagy, causing an accumulation of intracellular NFTs and extracellular Aβ plaques in AD brains. Degenerating neurons secrete pathological tau into the extracellular space, where healthy neurons, microglia, astrocytes, OPC, and OL can endocytose/phagocytose tau for clearance. However, secreted tau species, including monomers, oligomers, and PHFs, are seeding competent, which propagates tau pathology in neurons and mature OL. Degenerating neuronal dendrites can secrete CSF1 and IL-34 that induce replicative senescence in microglia associated with the extracellular Aβ plaques (1). Pathological tau induces proteotoxic stress-driven senescence in microglia upon proteostasis impairment (2). Endocytosed tau oligomers can induce senescence in astrocytes and lead to the secretion of HMGB1 into the extracellular space. Senescent astrocytes may impair glutamate homeostasis, leading to glutamate excitotoxicity in AD (3). Protein aggregates (PAs) and senescent astrocytes secreted HMGB1 can also impair the differentiation of OPC into mature OL, causing demyelination of neurons (4). Abbreviations: CSF1, colony-stimulating factor 1; IL-34, interleukin-34; PAs, protein aggregates; Aβ, amyloid beta; NFT, neurofibrillary tangle; OPC, oligodendrocyte progenitor cell; OL, oligodendrocyte; and HMGB1, high mobility group box 1.

Synaptic plasticity is crucial for memory, which has been hypothesized to be stored as spines at the postsynaptic dendrites and maintained by the regulated firing of neurotransmitters and persistent firing of action potentials or spikes from presynaptic neurons [91,92]. The persistent or spontaneous firing of neurons is essential for working memory [93,94], which is affected in AD patients [95,96]. Interestingly, AD patient-derived induced neurons (iNs) overexpressing p16, a cell cycle regulatory protein, induces senescence and eventually loses many neuronal characteristics, including a decrease in spontaneous firing and long-term potentiation (LTP) and increased long-term depression (LTD), causing decreased synaptic transmissions [83,97]. Interestingly, 97% of senescent cells in the AD brain appear to be excitatory neurons positive for elevated senescence marker p19 and NFTs based on single-cell analyses [98]. Notably, NFTs-bearing neurons express apoptotic markers, including activated caspase-3, propidium iodide (PI)-incorporated DNA, and loss of membrane integrity in the brains of rTg4510 mouse models of tauopathy [77]. However, despite having an apoptotic phenotype, the neurons survive longer, and perhaps NFTs drive neuronal senescence and likely delay apoptosis [77]. Another study has shown the co-occurrence of NFT burden and senescence-like phenotypes, including an elevated expression of the cell cycle, survival, inflammation, NF-κB, and SASP, and decreased levels of cell death-related pathway proteins in brains of human AD and rTg4510 mice via gene expression profiling of laser capture-microdissected cortical neurons [81]. Treating the rTg4510 transgenic mice with senolytic drugs, dasatinib (D) + quercetin (Q) effectively ameliorated NFT burden and SASP by killing senescent cells. It restored the brain structure, suggesting intracellular tau aggregates driven neuronal senescence [81]. The degenerating proteostasis-compromised senescent neurons can secrete tau in the forms of monomers, oligomers, and PHFs into the extracellular space, which is then taken up by the healthy neurons or non-neuronal cells that lead to the spreading of pathological tau (Figure 6).

### 3.3. Senescence in Astrocytes

Astrocytes are neuronal-supporting proliferative glial cells and play an important role in regulating neuronal synaptic plasticity by maintaining glutamate homeostasis. Astrocytes express excitatory amino acid transporters, EAAT1/2 and glutamine synthase (GS), to recycle excess glutamate released by the presynaptic neurons and catabolize into glutamine, which is transported back to the neurons for glutamate synthesis. Thus, astrocytes prevent glutamate-associated excitotoxicity in neurons [99,100]. Nevertheless, the glutamate homeostasis pathway is dysregulated with age and in AD brains by decreasing the levels of GS and EAAT1/2 in astrocytes [101,102] and extrasynaptic retention of glutamate, causing glutamate excitotoxicity [103].

The presence of senescence markers p16 and matrix metalloproteinase-1 (MMP-1) in astrocytes have been identified in the frontal cortex of human autopsy AD brains, and upon treatment with Aβ_1–42_ oligomers, suggesting that astrocytes are also senescent in human AD brains [104]. Senescence induced by X-irradiation (IR) in human astrocytes shows a decreased expression of EAAT1/2 and voltage-gated potassium channel protein Kir4.1, and water transport channel protein AQP4, and an increased expression of APOE and connexin-43. Notably, co-culturing of senescent astrocytes with fetal human cortical neurons causes glutamate excitotoxicity, which suggests that cellular senescence is not unique to AD but also in other contexts of glutamate excitotoxicity such as epilepsy, ALS, and Huntington’s disease [105].

**Figure 7:**
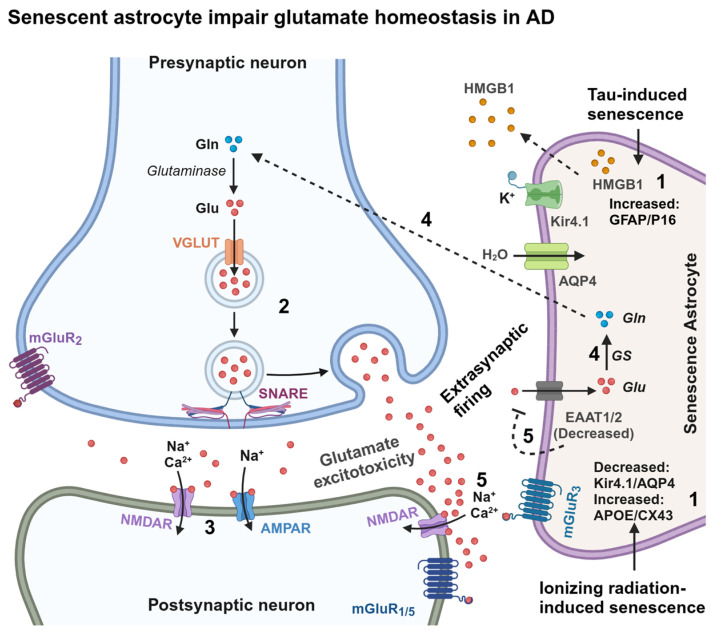
***Senescent astrocytes impair glutamate homeostasis in AD*.** Senescence in astrocytes is induced by AD-associated tau oligomers/Aβ_1–42_ peptide. Senescent astrocytes show characteristics of increased GFAP, p16, HMGB1, APOE, and connexin-43 (CX43), and decreased EAAT 1/2, Kir4.1, and AQP4 levels upon exposure to ionizing radiation (1). The glutamate–glutamine cycle is essential for proper neuronal excitation by which presynaptic neurons release glutamate at the synaptic zone. The SNARE complex regulates the fusion of glutamate vesicles with the presynaptic membrane to release glutamate (2). AMPAR/NMDAR binds to glutamate, eventually allowing an influx of Na+ and Ca^2+^ ions to induce an action potential at the postsynaptic neurons (3). Excessively released glutamate is taken up by astrocytes via EAAT 1/2 channels, where glutamine synthase (GS) converts glutamate into glutamine and is exported into neurons for the next cycle of neuron excitation by which regulated neurotransmission is maintained (4). In senescent astrocytes, the EAAT 1/2 level is decreased, causing the accumulation of glutamate in the synaptic cleft and extrasynaptic regions, which leads to excitotoxicity (5), impairing AD memory retrieval. Abbreviations: HMGB1, high mobility group box 1; GFAP, glial fibrillary acidic protein; p16, cyclin-dependent kinase inhibitor 2A; K^+^, potassium ion; Kir4.1, inwardly rectifying potassium channel 4.1; AQP4, aquaporin-4; GS, glutamine synthase; EAAT 1/2, excitatory amino acid transporters 1/2; APOE, apolipoprotein E; CX43, connexin-43; Gln, glutamine; Glu, glutamic acid; VGLUT, vesicular glutamate transporter; SNARE, soluble N-ethylmaleimide-sensitive factor attachment protein receptor; mGluR, metabotropic glutamate receptor; Na^+^, sodium ion; Ca^+^, calcium ion; AMPAR, alpha-amino-3-hydroxy-5-methyl-4-isoxazolepropionic acid receptor; and NMDAR, N-methyl-D-aspartate receptor.

Astrocytes are sensitive to oxidative stress and proteotoxic stresses induced by exogenous hydrogen peroxide H_2_O_2_ and epoxomicin/lactacystin, respectively, and upon chronic exposure, induce senescence [106]. Notably, the co-occurrence of tau oligomers and senescent marker p16 in the reactive astrocytes in the frontal cortex of human autopsy AD and frontotemporal dementia (FTD) brains indicate that astrocytes are also senescent in AD/AD-related dementias (ADRDs). Inhibiting the nuclear protein high mobility group box 1 (HMGB1, an alarmin) secretion into the extracellular space ameliorates the paracrine spreading of a senescent-like phenotype in the mouse primary astrocytes culture. Then, injecting HMGB1 secretion inhibitors in hTau mice brains attenuates tau pathology, neuroinflammation, and cognitive impairment [107]. Thus, senescent astrocytes may impact homeostasis at the tripartite synapse by dysregulating the glutamate balance, causing glutamate excitotoxicity, and likely triggering local neuroinflammation via molecules like HMGB1 (Figure 6 and Figure 7).

### 3.4. Senescence in Oligodendrocytes

Oligodendrocytes (OLs) are terminally differentiated myelinating cells that enwrap many axons by forming myelin sheaths, which are important for proper saltatory conduction [108,109]. Myelination is dysregulated (in other words, white matter damage) in various neurological diseases, including multiple sclerosis (MS) [110,111,112], ALS [113], and tauopathy [114]. The CNS-residing oligodendrocyte progenitor cells (OPCs) serve as stem cells for differentiating into mature OLs, which express tau at a relatively lower level than the neurons, perhaps after myelinating the axons [115]. Tau knockout mice show normal OL maturation and associated myelination on the axons, suggesting tau is dispensable for OL myelination in normal conditions [115,116]. But, the role of tau in OL maturation is controversial since in vitro studies show the downregulation of tau impairs the trafficking of myelin basic protein (MBP) mRNA granules and outgrowth defects most likely by a decreased interaction of α-tubulin with Fyn kinase, which can recruit microtubules (MTs) to the plasma membrane for OL outgrowth [117]. However, tau level is upregulated in OL and impairs its myelinating function in proteolipid protein-1 (PLP1), overexpressing transgenic mice of chronic hypomyelinating leukodystrophies (HDLs) [118].

The late-onset AD (LOAD) susceptible gene *BIN1* encoding protein BIN1 is predominantly expressed in mature OL around the white matter tracts [119]. In AD brains, BIN1 isoforms are differentially expressed with increased BIN1:H and decreased BIN1:L isoforms [119]. Tau has been shown to interact with BIN1, whereas AD-associated phosphorylated tau T231 decreases interaction with BIN1 [120]. However, it is unknown whether tau–BIN1 interactions regulate OL-mediated myelination of axons. The demyelinating neurons have been shown to recruit and activate OPCs by the spontaneous release of glutamate to differentiate into mature OLs for myelination, which is likely compromised in AD [121]. It is well known that the spontaneous release of glutamate at the synapse is essential for working memory, which is dysregulated in AD. Perhaps senescent astrocytes impair glutamate recycling, affecting OL-mediated remyelination, causing the loss of white matter tracts and decreased brain volume in AD. Notably, tau-associated memory impairment has been shown in the THY-Tau22 tauopathy model in which AT8-positive tau spread from hippocampal pyramidal neurons into OL, causing a loss of high-firing neural cells and neurodegeneration in aged mice [122].

Tau is a neuronal protein, but pathological tau aggregates are evident in non-neuronal cells of certain tauopathy. For instance, tau aggregates are mainly present in the neurons of AD brains. In contrast, in Progressive supranuclear palsy (PSP) and Corticobasal degeneration (CBD), tau aggregates are present in neurons, astrocytes, and OLs. Pathological tau isolated from AD, PSP, and CBD brains have been shown to propagate tau aggregates in OL in a cell-autonomous manner. However, tau pathology does not propagate in astrocytes, suggesting endogenous tau in mature OL contributes to tau propagation in mice [123]. Tau expression in OL is neural activity-dependent and requires remodeling/repairing the myelin sheath. Interestingly, OL has also been shown to generate amyloidogenic Aβ_1–42_ peptide and substantially contribute to amyloid β plaque formation without excitatory neuron-derived Aβ peptide in AD mice [124].

Notably, Aβ plaque-associated OPCs display senescence-like phenotypes in the brains of human AD and APP/PS1 transgenic mice, with the latter showing toxic effects of oral administration of senolytic drugs D + Q on senescent OPCs [125]. It is possible that chronic exposure to OPCs with Aβ or tau aggregates impairs proteostasis activity and may promote senescence in OPCs and inhibit differentiation into mature OLs, causing loss of white matter tracts in AD and other tauopathy. Alternatively, HMGB1 from tau oligomer-exposed senescent astrocytes [107] may impair OPC differentiation into mature OLs. To support the second hypothesis, senescent factor HMGB1 has been shown to impair OPC differentiation into OLs in the lysolecithin-injected spinal cord demyelinating mouse model in which HMGB1 binds to the receptor Toll-like receptor 2/1 (TLR 2/1), activates NF-κB signaling, and eventually causes impaired CNS remyelination [126] (Figure 6). To conclude this section, protein aggregates can drive senescence in oligodendrocytes. Furthermore, senescence in OPCs may impair the differentiation of OPCs into OLs and consequently dysregulate OL-mediated myelination on axons.

### 3.5. Senescence in Microglia

Microglia are immune cells of the CNS that function like macrophages and maintain the homeostasis of neurons, astrocytes, and oligodendrocytes. Microglia are proliferated upon CNS injury, exposure to protein aggregates, and viral infections. The primary defensive response of microglia is to clear away the dead cells, cell debris, damage-associated molecular patterns (DAMPs), and viruses by phagocytosis or endocytosis and inducing chemotaxis via releasing inflammatory cytokines, chemokines, and growth factors. However, chronic microglial activation can adversely affect neurons and non-neuronal cells in the CNS. For example, Down’s syndrome (DS) is caused by trisomy of human chromosome 21, where the AD risk gene (amyloid precursor protein or APP) resides. In DS, increased type I interferon receptors (IFNARs) in microglia, synaptic pruning, and microglial senescence, which may be in response to amyloid and/or tau pathologies, have been observed [127,128,129]. Thus, it suggests that AD/DS-related pathological changes trigger microglial senescence and may lead to neuroinflammation.

Notably, increased synaptic pruning has been shown to impair memory before Aβ deposition in the J20 transgenic AD mouse model [130]. The soluble Aβ oligomers activate microglia and eventually eliminate the synapses excessively via complement receptor 3 (CR3). The inhibition of complement pathway-associated proteins C1q and C3 or the CR3 receptor decreased the number of phagocytic microglia and inhibited Aβ oligomer-induced synapse loss [130]. The transmembrane immune signaling adaptor protein TYROsine kinase Binding Protein (TYROBP), otherwise known as DNAX-Activation Protein 12 (DAP12), has been shown to associate with various microglial receptors, including Triggering Receptor Expressed on Myeloid Cells 2 (TREM2) [131], CD33 [132], CR3 [133], and SIRPβ1 [134], which are either differentially expressed or genetic risk factors for LOAD. Deficiency of DAP12 in tauopathy PS19 and amyloidogenic APP/PS1 mice alleviates cognitive impairment via attenuating the C1q-CR3 complement pathway despite increased tau pathology and diffused Aβ plaques, respectively [135,136,137]. However, loss of TREM2 has been shown to exacerbate axonal dystrophy around the Aβ plaques [138]. Senolytic drugs specifically eliminated TREM2 expressing senescence microglia, but not disease-associated microglia (DAM) [139] suggesting that TREM2 may likely engage different transmembrane adaptor proteins depending on the context of disease progression.

Microglia can take up monomeric and fibrillar Aβ_1–42_ for clearance via receptor β1 integrin-mediated phagocytosis and micropinocytosis, respectively [140,141]. Microglia can also be recruited to the Aβ plaques, where microglia function as a physical barrier to prevent the seeding of soluble Aβ_1–42_ into Aβ plaque. In addition to microglial barrier function to compactly packed amyloid plaques [142], reactive microglia can also phagocytose Aβ oligomers for clearance [140,141,143] and secrete enzymes to proteolyze Aβ plaque [144]. But then, how microglia survive despite functioning as a physical barrier to Aβ plaque and internalizing large amounts of Aβ oligomers chronically is still unclear. It is possible that degenerating neurons can release macrophage colony-stimulating factor 1 (CSF1 or M-CSF) and IL-34, which can accelerate the microglial proliferation capacity to recruit newly formed microglia to the plaques and eventually reach the Hayflick limit, causing replicative microglial senescence in AD [145,146,147,148]. However, it is unclear whether the Hayflick senescent microglia retained their barrier function against the amyloid plaques in AD (Figure 6), i.e., the likelihood of senescent microglia continuing to phagocytose amyloid is unclear.

Microglia have been shown to survive longer durations, from 15 months to the entire mouse lifespan, without undergoing proliferation [149]. However, in the APP/PS1 mouse model of AD, microglia shows accelerated proliferation that arises from matured microglia and migrates to the Aβ plaque, indicating that repeated cell division can induce replicative microglial senescence in AD [149]. Aβ plaque-associated microglia often display the DAM phenotype, with an upregulated expression of TREM2 and C-type lectin domain containing 7A (CLEC7A) and elevated autophagy to clear protein aggregates [150]. However, microglial autophagy is impaired upon chronic exposure to protein aggregates, which alters the cellular metabolism and induces senescence. A previous study has shown that the genetic deletion of autophagy regulatory genes (ATG7 and ATG14) in 5xFAD transgenic mice induced microglial senescence. Treating these mice with senolytic drugs D + Q eliminated the senescent microglia and ameliorated AD pathology [151,152]. This suggests that autophagy deficiency may promote proteotoxic-driven microglial senescence in AD.

In addition to Aβ plaque, tau can induce microglial senescence and impair microglial motility in mouse primary microglial cells [153]. Aging can also induce microglial senescence around the white matter in the brain and spinal cord, causing neuroinflammation-associated demyelination in the white matter axons [154]. Thus, it is suggested that protein aggregates or aging can trigger microglial senescence. The novel senescent reporter INK-ATTAC (p16^Ink4a^-apoptosis through targeted activation of caspase 8) transgenic mice have been used to eliminate the senescent cells by activating apoptosis using a synthetic drug AP20187, which induces the dimerization of membrane-bound enhanced green fluorescent protein (EGFP) tagged-myristoylated FK506 binding protein (EGFP-FKBP)-Caspase 8 fusion proteins under the control of the p16 (INK4a) promoter that is designed to be activated in senescent cells expressing p16 (INK4a) [155]. Pathological tau can induce senescence in the brains of senescent reporter INK-ATTAC mice when crossed with PS19 transgenic mice. Notably, the administration of AP20187 from weaning age ameliorated tau pathology and improved cognition by reducing the number of senescent microglia and astrocytes [156]. Furthermore, eliminating senescent cells in the whole body by administering AP20187 or D + Q intraperitoneally in aged INK-ATTAC transgenic mice shows attenuated aging-associated brain inflammation and cognitive impairment that implies eliminating senescent cells restores homeostasis in the brain [157]. Thus, protein aggregates in AD or tauopathy brains can trigger senescence in microglia by the Hayflick limit (proliferative/replicative senescence) and proteotoxic stress driven by impaired UPS and autophagy (Figure 6).

### 3.6. Replicative Senescence

A bioinformatics profiling of mRNA transcripts in multiple single nuclei using a human normal aging, AD, and Parkinson’s disease-associated Lewy body dementia (PD-LBD) brains dataset suggests the presence of cell cycle regulatory mRNA transcripts such as G2/M-phase marker cyclin B and S-phase marker proliferating cell nuclear antigen (PCNA) in excitatory neurons [158]. These cell cycle markers and senescent neurons are abundant in sporadic AD brains but not normal aging brains [158]. However, the accelerated proliferation rate and induction of replicative senescence were observed in Aβ plaque-associated microglia in AD [145]. Most likely, the degenerating neurons are transcriptionally activating the expression of cell cycle regulatory proteins. However, protein aggregates may prevent neurons from entering or completing the entire cell cycle, which is impossible for terminally differentiated cells like neurons. Instead, it switches to a senescent phenotype. It is unclear how protein aggregates regulate the post-mitotic neurons to become senescent. Still, they may induce complex molecular changes in the pro-senescence transcript(s), which triggers senescence. Thus, it is motivating to speculate the hypothesis that the intracellular accumulation of NFTs can drive neuronal senescence by proteotoxic stress. In contrast, extracellular Aβ plaques drive replicative senescence in mitotic microglia in the early stage of AD, before the beginning of tau pathology. Once tau pathology sets in, tau released from degenerating senescent neurons can trigger proteotoxic stress-induced senescence in microglia. Thus, both replicative and proteotoxic-driven senescent microglia may exacerbate SASP secretion in addition to senescence in OPCs and astrocytes, causing neuroinflammation and eventually neurotoxicity, leading to decreased brain volume in AD (Figure 6).

## 4. Proteotoxic Stress Drives Cellular Senescence

### 4.1. Proteasome in Senescence

A tightly regulated protein homeostasis is essential for healthy cell survival. The mammalian target of rapamycin (mTOR) signaling-associated protein synthesis and molecular chaperone-assisted protein folding are critical for protein function. Protein degradation by the UPS and AMP-activated protein kinase (AMPK) signaling-associated autophagy ensures the quality of the functional proteins. However, various factors, including cellular stress, aging, sporadic and inherited metabolic and neurodegenerative diseases, and infectious disease, dysregulate protein homeostasis, causing proteotoxic stress, which drives cellular senescence. The UPS and molecular chaperones are indispensable for regulating the protein homeostasis, which is deteriorated in senescent cells [159].

Proteasomal activity is impaired in aged animal tissues, including the skeletal muscle [160,161] and brain [162]. Likewise, tau oligomers and PHFs isolated from the autopsy of human AD brains impair proteasomal activity [16,163], which may trigger chronic proteotoxic stress, causing senescence in human AD brains. The direct role of impaired proteasomal or lysosomal activities inducing proteotoxic stress-associated premature senescence has been identified upon chronic treatment of human fibroblasts with the proteasomal inhibitor MG-132 [164,165,166,167] or lysosomal acidification inhibitor Bafilomycin A1 (Baf A1) [167]. Increased generation of reactive oxygen species (ROS) and accumulation of defective mitochondria were also found in Baf A1-driven senescence in human fibroblasts [167]. Interestingly, some of the proteasome subunits and proteasome-interacting proteins reduced at both the mRNA transcripts and protein levels, causing decreased proteasomal activity in replicative senescent human fibroblasts and animals’ aged muscle and brain tissues [160,162,164].

**Figure 8:**
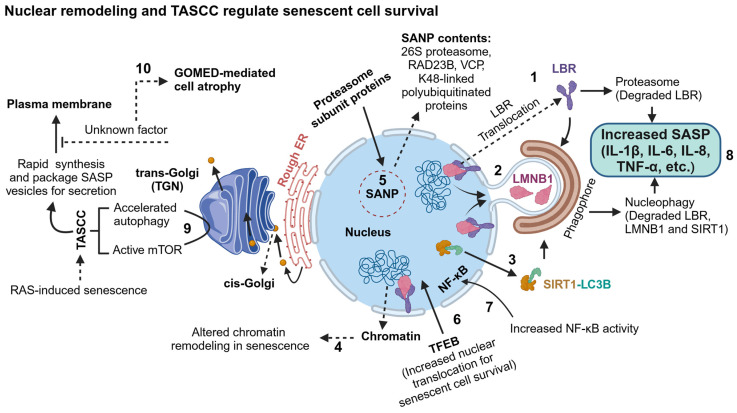
***Nuclear remodeling and TOR-autophagy spatial coupling compartment (TASCC) regulate senescence cell survival*.** Senescent-inducing factors regulate the translocation of nuclear transmembrane protein lamin B receptor (LBR) into the cytoplasm for proteasomal degradation or are, perhaps, degraded by nucleophagy (1). Chronic inhibition of the UPS or autophagy induces senescence by remodeling the nucleus for relaxed gene expression. The oncogene RAS-induced autophagy, but not by mTOR inhibition or starvation-induced autophagy, regulates the degradation of lamin B1 (LMNB1), causing oncogene-induced senescence (2). In senescent cells, nuclear autophagy (nucleophagy) degrades LMNB1 nuclear blebs (2) and SIRT1 (3) by interacting with ubiquitin-like LC3B to remodel the nuclear membrane and chromatin for relaxed gene expression (4). Senescent cells import the proteasome subunit proteins into the nucleus to assemble the 26S proteasome and eventually form senescence-associated nuclear proteasome foci (SANP) by actively recruiting VCP/p97 ATPase and RAD23 to degrade unknown nuclear proteins (5). Senescent cells enhance the nuclear translocation of active dephosphorylated TFEB (6) and NF-κB (7) for increased lysosome biogenesis and senescence-associated immune response gene expression (8), respectively. In RAS-induced senescence, the trans-Golgi network (TGN) provides a novel compartment, TOR-autophagy spatial coupling compartment (TASCC), for accelerated mTOR and autophagy activities (9), which is essential for senescent cell survival. An alternative autophagy, LC3-independent Golgi-membrane-associated degradation (GOMED) pathway is activated upon the impairment of the Golgi to plasma membrane trafficking that eventually triggers extensive remodeling of the nuclear membrane leading to the terminal cell atrophy (10). Abbreviations: LBR, lamin B receptor; LMNB1, lamin B1; SIRT1, sirtuin 1; LC3B, microtubule-associated protein 1 light chain 3 beta; TFEB, transcription factor EB; TGN, trans-Golgi network; SASP, senescence-associated secretory phenotype; NF-κB, nuclear factor kappa B; IL, interleukin; TNFα, tumor necrosis factor alpha; ER, endoplasmic reticulum; RAD23B, UV excision repair protein RAD23 homolog B; VCP, valosin-containing protein; mTOR, mammalian target of rapamycin; RAS, rat sarcoma virus; TASCC, TOR-autophagy spatial coupling compartment; GOMED, Golgi-membrane-associated degradation; and SANP, senescence-associated nuclear proteasome foci.

Notably, senescent cells form membrane-less nuclear proteasome foci, which are liquid droplets of 26S proteasome condensates induced by K48-linked ubiquitin chains and shuttling factor RAD23B. The senescence-associated nuclear proteasome foci (SANP) recruit the ubiquitin chaperone VCP/p97 ATPase to degrade K48-linked polyubiquitinated proteins in the nucleus. Inhibiting either ubiquitination or proteasomal activity and knocking down RAD23B decreases SANP formation causing increased mitochondrial respiration and ROS generation [168,169]. This finding suggests that the SANP drives senescence by degrading unknown proteins in the nucleus, and inhibiting SANP formation at later stages of senescence does not prevent the senescent characteristics (Figure 8). However, it is not clear how senescent cells survive upon impairment of proteasomal activity in AD and tauopathy brains.

### 4.2. Autophagy in Senescence

Proteotoxic stress drives cellular senescence to resist apoptosis, which can be induced by excessive activation or inhibition of autophagy. It is still unclear how protein aggregates dysregulate autophagy and become senescent cells, which are pro-inflammatory. Inhibiting autophagy at a later senescence stage does not affect senescent cell survival as they are found in many neurodegenerative diseases. The nuclear membrane may sense the proteotoxic stress in the cytoplasm and eventually trigger chromatin remodeling for transitioning into senescence. To support this hypothesis, autophagy has been shown to induce the degradation of nuclear cytoskeleton protein lamin B1 (LMNB1) and its receptor LBR and sirtuin 1 (SIRT1), which levels serve as senescent biomarkers (Figure 8).

The inner nuclear membrane-associated cytoskeleton protein lamins (lamin A, B1, B2, and C) regulate the nucleus’s stability, size and shape, and chromatin reorganization. Senescent cells are characteristics of enlarged and irregularly shaped nuclei with the loss of LMNB1, which occurred upon the overexpression of p16 and activation of either p53 or retinoblastoma protein (pRB) pathways. The loss of LMNB1 is independent of p38 mitogen-activated protein kinase (p38 MAPK), Ataxia telangiectasia mutated (ATM) serine/threonine kinase and NF-κB activation, and cellular ROS generation [170,171]. However, p38 MAPK has been shown to phosphorylate the stress kinase Unc-51-like autophagy-activating kinases (ULK1) S555 to induce autophagy-mediated senescence against chemotherapy-induced apoptotic-prone cells in cancer [172]. A direct role of autophagy-mediated degradation of nuclear blebs of LMNB1 by interacting with lipidated autophagy protein LC3-II (ATG8) has been shown in oncogene RAS-induced senescence but not in cases where autophagy is induced either by starvation or mTOR inhibition [173]. Autophagy inhibition via knockdown of autophagy regulatory proteins ATG7, ATG12, and LAMP2 also induces premature senescence through p53 and ROS pathways in primary human fibroblasts [174]. Moreover, LMNB1 dysfunction and its associated DNA heterochromatin relaxation have been identified in the *D. melanogaster* tauopathy model system and autopsy human AD brains, suggesting that tauopathies can be categorized into neurodegenerative laminopathies [175]. SUMOylated lamin A/C is also recognized by LC3 to induce autophagic degradation of both lamin A/C and escaped nuclear DNA in response to DNA damage [176].

The nuclear membrane protein lamin B receptor (LBR) regulates the nuclear heterochromatin organization, and its activity is downregulated in senescence. Human cells treated with proteasomal inhibitor MG-132 [177], DNA damage inducer γ-ray irradiation [178], and replicative senescent inducer thymidine [179] or BrdU [180] translocated the LBR into the nucleoplasm and cytoplasm for degradation, and thus, cells transition to senescence. Moreover, mutations in the *LMNA* gene have been shown to induce accelerated premature aging in Hutchinson–Gilford progeria syndrome (HGPS) [181,182]. SIRT1 is an NAD^+^-dependent deacetylase that has a broad range of substrates and, notably, functions as a sensor of the cytosolic NAD^+^/NADH ratio [183] and regulates histone deacetylation [184] and NF-κB activity [185] in the nucleus to repress gene expression. Notably, the presence of the LIR (LC3-interacting region) motif in SIRT1 supports the autophagic degradation of SIRT1 by directly interacting with LC3B to promote cellular senescence transition [186] (Figure 8).

Thus, excessive activation or inhibition of autophagy is detrimental to cells and consequently remodels the heterochromatin for relaxed gene expression before transitioning into senescence. Thus, nuclear remodeling in senescence is a cell survival mechanism by activating nucleophagy degradation of LMNB1, LBR, and SIRT1, and SANP-mediated degradation of unknown nuclear foci proteins. However, senescent cells are pro-inflammatory and negatively affect the healthy cell’s survival at a late stage of senescence or disease context and secrete a wide range of SASP components such as IL-1β, IL-6, IL-8, and TNF-α. Senescent cells resist apoptosis, continue growing, and produce enormous amounts of SASPs, which require active mTOR for increased protein translation. Interestingly, a distinct compartment has been identified in oncogene RAS (rat sarcoma virus)- induced senescent cells at the trans-Golgi network (TGN), which serve as a TOR-autophagy spatial coupling compartment (TASCC), where the active mTOR and autophagy work together to rapidly synthesis and package SASP contents in membranous vesicles from autophagic turnover for secretion [187]. In senescent cells, lysosomal acidification is impaired, and there is increased membrane permeability, lipofuscin accumulation, increased senescence-associated β-galactosidase (SA-β-gal) activity, and decreased proteolytic capacity [188]. However, increased nuclear translocation of dephosphorylated transcription factor EB (TFEB), a transcription factor, induces lysosome biogenesis, which is sufficient and compensates for the degradative capacity of defective lysosomes for senescent cell survival [189] (Figure 8). Thus, lysosomal activity is indispensable for senescent cell survival, and understanding the mechanism of the lysosomal biogenesis pathway in senescent cells could be a potential druggable pathway to develop a novel senolytic drug strategy to eliminate the senescent cells.

Autophagy dysfunction is a major hallmark of neurodegenerative disease. The final stage of AD is brain atrophy with extensive neuronal cell loss accompanied by decreased brain volume. However, the exact mechanism of brain atrophy is unknown. In AD brains, proteotoxic stress drives neuronal senescence, typically apoptosis-resistant. Nonetheless, cells die at a late stage of senescence by unknown mechanisms. Interestingly, the Golgi-membrane-associated degradation (GOMED) has been considered as an alternative autophagy that can function in the absence of LC3 (ATG8/autophagy-related protein 8) conjugation machinery, including ATG5, ATG7, and LC3. Notably, the other autophagy regulatory proteins, including ULK1, Beclin1, and Rab9 are essential for LC3-independent autophagy. The GOMED pathway is activated upon the impairment of the Golgi to plasma membrane trafficking, and eventually, the Golgi membrane generates the double-membranous autophagosomes [190,191]. Moreover, an early-onset of autolysosomal pathology has been identified in the polyglutamine disease dentatorubral-pallidoluysian atrophy (DRPLA), in which canonical autophagy is chronically inhibited and eventually activates the alternative autophagy pathways, including the GOMED and nucleophagy-mediated degradation or secretion of LMNB1. Thus, LC3-independent alternative autophagy extensively disrupts the nuclear membrane integrity, causing neuronal degeneration, eventually leading to terminal cell atrophy [192] (Figure 8).

## 5. Concluding Remarks

AD is a protein aggregation disease affecting the heterogeneous types of cells in the human brain, causing memory retrieval dysfunctions and loss of brain volume. Tauopathy is a group of more than 20 neurodegenerative diseases caused by aggregation of tau in neurons. AD is the most prevalent tauopathy associated with extracellular aggregation of Aβ plaques and intracellular accumulation of NFTs. Thus, understanding the molecular and cellular mechanisms of the dynamics of pathological tau degradation or clearance in AD will immensely benefit understanding the basics of tau degradation and its dysfunction in more than twenty tauopathies and eventually develop novel effective therapeutic strategies. Tau is a natively unfolded protein and does not have a definite structure. In AD, phosphorylated tau is misfolded and eventually recruits molecular chaperones Hsc70/HSP70 and HSP90 and their co-chaperones BAG-1 and FKBP51 to prevent tau aggregation or disease progression. Multiple degradative systems, including the 20S proteasome, 26S proteasome, CMA, autophagy, and aggrephagy control the tau level in neurons. If so, then it is still unclear how neurons determine which degradative pathway needs to be activated to maintain tau at the physiological level. Post-translational modifications on tau, such as phosphorylation, acetylation, and ubiquitination, determine which degradative pathway is required to clear pathological tau. The 20S proteasome degrades natively unfolded tau, whereas CMA degrades folded tau that can be phosphorylated or non-phosphorylated. The 26S proteasome degrades phosphorylated tau conjugated with ubiquitin chains, mainly K48-linked polyubiquitinated. Furthermore, 26S may also degrade hybrid ubiquitinated tau such as K48/K6-, K48/K11-, and K48/K63-linked polyubiquitin chains not identified in tau. Macroautophagy (autophagy) degrades the impaired 26S proteasome substrate of tau, which is phosphorylated, acetylated, and ubiquitinated. Tau aggregates form upon the sequential failure of proteasome and autophagy. Aggrephagy specifically degrades tau aggregates, and NFTs form upon the impairment of aggrephagy. The ubiquitin chaperone complex VCP-HSP70 extracts pathological tau (monomers, oligomers, and PHFs) from NFTs for either the UPS or autophagy-mediated tau clearance, which is impaired in neurons and eventually secretes pathological tau into the extracellular space (Figure 2). Neurons become senescent to prevent adverse effects from accumulating NFTs and the sequential failure of protein degradation pathways. Secreted tau can be cleared by healthy neurons and their supporting cells, including astrocytes, microglia, OPCs, and OLs. However, pathological tau impairs proteostasis in the recipient cells and may turn them into senescent cells. Chronically inhibiting autophagy with Baf-A1 and the 26S proteasome with MG-132 is sufficient to induce senescence in human fibroblasts, indicating that proteotoxic stress drives senescence. Microglia are guardians of the CNS cell types and receive signals from neurons, astrocytes, and OLs to maintain homeostasis. However, microglia become hypertrophic senescent cells due to chronic exposure to stress signals from other cell types and eventually secrete various SASP contents into the extracellular space. Secreted SASP from different cell types can adversely affect the CNS environment, causing extensive neuronal cell death and brain atrophy (Figure 6). Thus, designing novel drugs targeting both clear tau aggregates and alleviating senescent cells associated with immune responses will be a potential therapy for tauopathy, including Alzheimer’s disease.

## 6. Challenges and Future Directions

In this review article, we briefly described the basic mechanisms of tau degradation and its link with post-translational modifications that precede sequential tau degradation in AD brains. Since AD is a chronic brain illness, impairment in tau degradation causes proteotoxic stress, which signals the nucleus to undergo remodeling and trigger apoptosis-resistant senescent phenotypes for cell survival. Senescent cells are non-dividing, pro-inflammatory, defective in protein degradation systems, and survive through secretory pathways by secreting various SASP factors. The secreted SASPs can adversely affect the proteostasis and cell homeostasis activities in adjacent cells, triggering more senescence and cell death, reducing the brain volume, and spreading tau pathology in AD. However, the exact mechanism of how UPS and autophagy are impaired in AD brains, causing the accumulation of tau aggregates and triggering downstream effects, is unknown.

Notably, a genome-wide association study (GWAS) provides rich information about the genetic markers associated with AD. Most human genetic mutations associated with tau pathology directly or indirectly affect the protein degradation systems, causing proteotoxic stress, eventually exacerbating oxidative and nuclear stress, including impaired DNA damage response, thus promoting senescence. Therefore, functional characterization of AD risk factor genes relevant to proteostasis activity will help understand how they affect the UPS and autophagy activities causing the accumulation of pathological tau. Interestingly, some AD risk genes (*APOE*, *BIN1*, *PICALM*, *CD2AP*, *SORL1*, *GRN*, *PLD*, *PLD3*, *INPP5D*, *MEF2C*, *EPHA1*, *PTK2B*, *CLU*, and *TREM2*) are directly or indirectly associated with endosome–lysosome and autophagy pathways and regulate tau degradation (reviewed elsewhere [14,193,194]). Tau has also been shown to accumulate in various human gene mutations associated with tauopathy brains [195] such as frontotemporal dementia (FTD)-associated *CHMP2B* [196,197,198,199], *UBQLN2* [200,201,202,203], *VCP* [204] and *SQSTM1* [205,206,207,208], and Parkinson’s disease (PD)-associated *LRRK2* [209,210] and Pick’s disease (PiD), PSP, CBD-associated *MAPT* haplotypes [211,212,213], and ALS-associated *C9orf72* [214] and TDP-43 [215,216]. Thus, it is clear that AD risk genes and mutations in other tauopathy-associated genes impair proteostasis, causing proteotoxic stress and tau accumulation.

Since the tau clearance mechanism is important for preventing AD progression, it will be helpful to develop an effective therapeutic strategy against the accumulation of pathological tau by understanding the basic mechanism of how proteostasis activity is impaired by AD risk genes. There are some small molecules that can enhance autophagy-mediated tau degradation and also prevent tau transmission, as reviewed elsewhere [14]. However, some drugs may not work effectively because of the presence of senescent cells in AD brains. The future therapeutic strategies for AD or tauopathy could be firstly eliminating senescent cells using senolytic drugs followed by strategies to clear tau and promote anti-inflammatory effects, which will be a potential therapy to prevent the disease progression. There will be some challenges in understanding the impact of drugs in the above-mentioned therapeutic strategies because of the need for standard biomarkers to diagnose proteotoxic stress and senescence at the cerebrospinal fluid (CSF) or blood levels in the patients. There are many senotherapeutics currently available that include the killing of senescent cells by senolytics targeting the anti-apoptotic BCL-2 protein family and decreasing the level and activity of SASP factors by senomorphics as reviewed elsewhere [86,217,218]. Since AD is a multifactorial disease, in addition to senotherapeutics, strategies to enhance pathological tau clearance are essential as combinatorial therapy for AD.

Identifying senescent cell-secreted SASP markers in the CSF or blood levels may be challenging because some of the cytokines overlap with NF-κB-activated innate immune responses in the cells, such as DAMs, which are different from senescent microglia. It is important to identify a unique and common receptor at the cell surface level for senescent cells, which can be further sorted out based on the cell type marker in the brain or in vitro conditions. Sorted-out cells based on the above features can be further separated based on the type of SASP factors they secrete to distinguish them from other cell phenotypes, such as DAM and cell types (neurons, astrocytes, oligodendrocytes, and microglia), at least for in vitro conditions. There are many common senescent markers available to identify senescence at the cellular level, including the accumulation of lipofuscin, SA-β-gal in the lysosomes, and increased levels of p16, p19, p21, phosphorylated p53, and retinoblastoma protein, and the absence of cell proliferation marker Ki-67. These cellular senescence markers can be combined with cell-type specific markers to distinguish the cell types undergoing senescence in the brains. However, these cellular senescence markers may not be detectable in a living AD patient’s CSF or blood. Thus, identifying novel SASPs is crucial for distinguishing patient populations that may be responsive to senolytic intervention for AD.

The UPS degrades proteins, whereas autophagy degrades both proteins and intracellular defective organelles to keep the cells functioning. In AD, the UPS and autophagy are impaired, causing the accumulation of pathological tau, which eventually affects the endoplasmic reticulum-associated protein degradation (ERAD) pathway [219] and activates the unfolded protein response (UPR) signaling [220,221,222]. In another study, the accumulation of pathological tau did not induce the UPR in the rTg4510 mouse model of tauopathy [223]. This is in contrast to other prior studies, which showed an induction of UPR by pathological tau. The reason for the difference in ER stress–tau pathology in different studies using different tau models is still being determined. Chronic activation of the UPR induces cell apoptosis [222]. However, it is not clear how pathological tau dysregulates the UPR and induces senescence in AD brains.

Intracellular accumulation of pathological tau as a proteotoxic stress marker and a decreased level of lamin B as a cellular senescence marker for nuclear remodeling can help identify the causal link between proteotoxic stress and senescence in AD or tauopathy. However, whether senescent cells also undergo cell death pathways later in disease progression is unclear. Moreover, the balance between cell survival via senescence versus cell death by apoptosis or other mechanisms and how it aligns with brain atrophy in AD is unknown. Thus, understanding the mechanisms of how tau dysregulates the proteotoxic stress pathways, including the UPR, UPS, autophagy and apoptosis, and nuclear remodeling for senescent cell survival, tau pathology progression, and brain atrophy are critical to developing novel therapeutic strategies against senescent cell, tau accumulation, and brain atrophy in AD. It is essential to understand the dysfunction of the tau clearance mechanism and proteotoxic stress pathways in driving senescence/inflammation. Such information will help identify novel biomarkers linking tau pathology and senescence in AD brains.

## References

[1] Wang Y., Mandelkow E. (2016). Tau in physiology and pathology. Nat. Rev. Neurosci..

[2] LoPresti P. (2018). Tau in Oligodendrocytes Takes Neurons in Sickness and in Health. Int. J. Mol. Sci..

[3] Poorkaj P., Bird T.D., Wijsman E., Nemens E., Garruto R.M., Anderson L., Andreadis A., Wiederholt W.C., Raskind M., Schellenberg G.D. (1998). Tau is a candidate gene for chromosome 17 frontotemporal dementia. Ann. Neurol..

[4] Ghetti B., Oblak A.L., Boeve B.F., Johnson K.A., Dickerson B.C., Goedert M. (2015). Invited review: Frontotemporal dementia caused by *microtubule-associated protein tau* gene (*MAPT*) mutations: A chameleon for neuropathology and neuroimaging. Neuropathol. Appl. Neurobiol..

[5] Zhang Y., Wu K.-M., Yang L., Dong Q., Yu J.-T. (2022). Tauopathies: New perspectives and challenges. Mol. Neurodegener..

[6] Lantero-Rodriguez J., Camporesi E., Montoliu-Gaya L., Gobom J., Piotrowska D., Olsson M., Burmann I.M., Becker B., Brinkmalm A., Burmann B.M. (2024). Tau protein profiling in tauopathies: A human brain study. Mol. Neurodegener..

[7] Glenner G.G., Wong C.W. (1984). Alzheimer’s disease: Initial report of the purification and characterization of a novel cerebrovascular amyloid protein. Biochem. Biophys. Res. Commun..

[8] Masters C.L., Simms G., Weinman N.A., Multhaup G., McDonald B.L., Beyreuther K. (1985). Amyloid plaque core protein in Alzheimer disease and Down syndrome. Proc. Natl. Acad. Sci. USA.

[9] Grundke-Iqbal I., Iqbal K., Quinlan M., Tung Y.C., Zaidi M.S., Wisniewski H.M. (1986). Microtubule-associated protein tau. A component of Alzheimer paired helical filaments. J. Biol. Chem..

[10] Wood J.G., Mirra S.S., Pollock N.J., Binder L.I. (1986). Neurofibrillary tangles of Alzheimer disease share antigenic determinants with the axonal microtubule-associated protein tau (tau). Proc. Natl. Acad. Sci. USA.

[11] Kosik K.S., Joachim C.L., Selkoe D.J. (1986). Microtubule-associated protein tau (tau) is a major antigenic component of paired helical filaments in Alzheimer disease. Proc. Natl. Acad. Sci. USA.

[12] Lee M.J., Lee J.H., Rubinsztein D.C. (2013). Tau degradation: The ubiquitin-proteasome system versus the autophagy-lysosome system. Prog. Neurobiol..

[13] Chesser A.S., Pritchard S.M., Johnson G.V.W. (2013). Tau Clearance Mechanisms and Their Possible Role in the Pathogenesis of Alzheimer Disease. Front. Neurol..

[14] Jiang S., Bhaskar K. (2020). Degradation and Transmission of Tau by Autophagic-Endolysosomal Networks and Potential Therapeutic Targets for Tauopathy. Front. Mol. Neurosci..

[15] Ciechanover A., Kwon Y.T. (2017). Protein Quality Control by Molecular Chaperones in Neurodegeneration. Front. Neurosci..

[16] Puangmalai N., Sengupta U., Bhatt N., Gaikwad S., Montalbano M., Bhuyan A., Garcia S., McAllen S., Sonawane M., Jerez C. (2022). Lysine 63-linked ubiquitination of tau oligomers contributes to the pathogenesis of Alzheimer’s disease. J. Biol. Chem..

[17] Lasagna-Reeves C.A., Castillo-Carranza D.L., Sengupta U., Sarmiento J., Troncoso J., Jackson G.R., Kayed R. (2012). Identification of oligomers at early stages of tau aggregation in Alzheimer’s disease. FASEB J..

[18] Wesseling H., Mair W., Kumar M., Schlaffner C.N., Tang S., Beerepoot P., Fatou B., Guise A.J., Cheng L., Takeda S. (2020). Tau PTM Profiles Identify Patient Heterogeneity and Stages of Alzheimer’s Disease. Cell.

[19] Li L., Jiang Y., Wang J.Z., Liu R., Wang X. (2022). Tau Ubiquitination in Alzheimer’s Disease. Front. Neurol..

[20] Abi Habib J., Lesenfants J., Vigneron N., Van den Eynde B.J. (2022). Functional Differences between Proteasome Subtypes. Cells.

[21] Nixon R.A., Rubinsztein D.C. (2024). Mechanisms of autophagy–lysosome dysfunction in neurodegenerative diseases. Nat. Rev. Mol. Cell Biol..

[22] David D.C., Layfield R., Serpell L., Narain Y., Goedertà M., Spillantini M.G. (2002). Proteasomal degradation of tau protein. J. Neurochem..

[23] Ukmar-Godec T., Fang P., Ibáñez De Opakua A., Henneberg F., Godec A., Pan K.-T., Cima-Omori M.-S., Chari A., Mandelkow E., Urlaub H. (2020). Proteasomal degradation of the intrinsically disordered protein tau at single-residue resolution. Sci. Adv..

[24] Liu Y.H., Wei W., Yin J., Liu G.P., Wang Q., Cao F.Y., Wang J.Z. (2009). Proteasome inhibition increases tau accumulation independent of phosphorylation. Neurobiol. Aging.

[25] Sahu I., Mali S.M., Sulkshane P., Xu C., Rozenberg A., Morag R., Sahoo M.P., Singh S.K., Ding Z., Wang Y. (2021). The 20S as a stand-alone proteasome in cells can degrade the ubiquitin tag. Nat. Commun..

[26] Ryder B.D., Wydorski P.M., Hou Z., Joachimiak L.A. (2022). Chaperoning shape-shifting tau in disease. Trends Biochem. Sci..

[27] Shimura H., Schwartz D., Gygi S.P., Kosik K.S. (2004). CHIP-Hsc70 complex ubiquitinates phosphorylated tau and enhances cell survival. J. Biol. Chem..

[28] Petrucelli L., Dickson D., Kehoe K., Taylor J., Snyder H., Grover A., De Lucia M., McGowan E., Lewis J., Prihar G. (2004). CHIP and Hsp70 regulate tau ubiquitination, degradation and aggregation. Hum. Mol. Genet..

[29] Takayama S., Bimston D.N., Matsuzawa S., Freeman B.C., Aime-Sempe C., Xie Z., Morimoto R.I., Reed J.C. (1997). BAG-1 modulates the chaperone activity of Hsp70/Hsc70. EMBO J..

[30] Alberti S., Esser C., Höhfeld J. (2003). BAG-1—A nucleotide exchange factor of Hsc70 with multiple cellular functions. Cell Stress. Chaperones.

[31] Elliott E., Tsvetkov P., Ginzburg I. (2007). BAG-1 associates with Hsc70·Tau complex and regulates the proteasomal degradation of Tau protein. J. Biol. Chem..

[32] Weickert S., Wawrzyniuk M., John L.H., Rüdiger S.G.D., Drescher M. (2020). The mechanism of Hsp90-induced oligomerizaton of Tau. Sci. Adv..

[33] Blair L.J., Nordhues B.A., Hill S.E., Scaglione K.M., O’Leary J.C., Fontaine S.N., Breydo L., Zhang B., Li P., Wang L. (2013). Accelerated neurodegeneration through chaperone-mediated oligomerization of tau. J. Clin. Investig..

[34] Dickey C.A., Kamal A., Lundgren K., Klosak N., Bailey R.M., Dunmore J., Ash P., Shoraka S., Zlatkovic J., Eckman C.B. (2007). The high-affinity HSP90-CHIP complex recognizes and selectively degrades phosphorylated tau client proteins. J. Clin. Investig..

[35] Dickey C.A., Yue M., Lin W.-L., Dickson D.W., Dunmore J.H., Lee W.C., Zehr C., West G., Cao S., Clark A.M.K. (2006). Deletion of the Ubiquitin Ligase CHIP Leads to the Accumulation, But Not the Aggregation, of Both Endogenous Phospho- and Caspase-3-Cleaved Tau Species. J. Neurosci..

[36] Cuervo A.M., Dice J.F. (2000). Regulation of lamp2a levels in the lysosomal membrane. Traffic.

[37] Cuervo A.M., Dice J.F. (1996). A receptor for the selective uptake and degradation of proteins by lysosomes. Science.

[38] Kaushik S., Cuervo A.M. (2018). The coming of age of chaperone-mediated autophagy. Nat. Rev. Mol. Cell Biol..

[39] Bourdenx M., Martín-Segura A., Scrivo A., Rodriguez-Navarro J.A., Kaushik S., Tasset I., Diaz A., Storm N.J., Xin Q., Juste Y.R. (2021). Chaperone-mediated autophagy prevents collapse of the neuronal metastable proteome. Cell.

[40] Massey A.C., Kaushik S., Sovak G., Kiffin R., Cuervo A.M. (2006). Consequences of the selective blockage of chaperone-mediated autophagy. Proc. Natl. Acad. Sci. USA.

[41] Wang Y., Martinez-Vicente M., Krüger U., Kaushik S., Wong E., Mandelkow E.-M., Cuervo A.M., Mandelkow E. (2009). Tau fragmentation, aggregation and clearance: The dual role of lysosomal processing. Hum. Mol. Genet..

[42] Caballero B., Bourdenx M., Luengo E., Diaz A., Sohn P.D., Chen X., Wang C., Juste Y.R., Wegmann S., Patel B. (2021). Acetylated tau inhibits chaperone-mediated autophagy and promotes tau pathology propagation in mice. Nat. Commun..

[43] Min S.-W., Cho S.-H., Zhou Y., Schroeder S., Haroutunian V., Seeley W.W., Huang E.J., Shen Y., Masliah E., Mukherjee C. (2010). Acetylation of tau inhibits its degradation and contributes to tauopathy. Neuron.

[44] Cohen T.J., Guo J.L., Hurtado D.E., Kwong L.K., Mills I.P., Trojanowski J.Q., Lee V.M.Y. (2011). The acetylation of tau inhibits its function and promotes pathological tau aggregation. Nat. Commun..

[45] Kim M.-S., Mun Y.-S., Lee S.-E., Cho W.-Y., Han S.-H., Kim D.-H., Yoon S.-Y. (2023). Tau acetylation at K280 regulates tau phosphorylation. Int. J. Neurosci..

[46] Abreha M.H., Dammer E.B., Ping L., Zhang T., Duong D.M., Gearing M., Lah J.J., Levey A.I., Seyfried N.T. (2018). Quantitative Analysis of the Brain Ubiquitylome in Alzheimer’s Disease. Proteomics.

[47] Nakayama Y., Sakamoto S., Tsuji K., Ayaki T., Tokunaga F., Ito H. (2019). Identification of linear polyubiquitin chain immunoreactivity in tau pathology of Alzheimer’s disease. Neurosci. Lett..

[48] Cripps D., Thomas S.N., Jeng Y., Yang F., Davies P., Yang A.J. (2006). Alzheimer disease-specific conformation of hyperphosphorylated paired helical filament-Tau is polyubiquitinated through Lys-48, Lys-11, and Lys-6 ubiquitin conjugation. J. Biol. Chem..

[49] Tan J.M.M., Wong E.S.P., Kirkpatrick D.S., Pletnikova O., Ko H.S., Tay S.-P., Ho M.W.L., Troncoso J., Gygi S.P., Lee M.K. (2008). Lysine 63-linked ubiquitination promotes the formation and autophagic clearance of protein inclusions associated with neurodegenerative diseases. Hum. Mol. Genet..

[50] French M.E., Koehler C.F., Hunter T. (2021). Emerging functions of branched ubiquitin chains. Cell Discov..

[51] Oikawa D., Sato Y., Ito H., Tokunaga F. (2020). Linear Ubiquitin Code: Its Writer, Erasers, Decoders, Inhibitors, and Implications in Disorders. Int. J. Mol. Sci..

[52] Jahan A.S., Elbæk C.R., Damgaard R.B. (2021). Met1-linked ubiquitin signalling in health and disease: Inflammation, immunity, cancer, and beyond. Cell Death Differ..

[53] Furthmann N., Bader V., Angersbach L., Blusch A., Goel S., Sánchez-Vicente A., Krause L.J., Chaban S.A., Grover P., Trinkaus V.A. (2023). NEMO reshapes the α-Synuclein aggregate interface and acts as an autophagy adapter by co-condensation with p62. Nat. Commun..

[54] Goel S., Oliva R., Jeganathan S., Bader V., Krause L.J., Kriegler S., Stender I.D., Christine C.W., Nakamura K., Hoffmann J.-E. (2023). Linear ubiquitination induces NEMO phase separation to activate NF-κB signaling. Life Sci. Alliance.

[55] Wu C.-J., Conze D.B., Li T., Srinivasula S.M., Ashwell J.D. (2006). Sensing of Lys 63-linked polyubiquitination by NEMO is a key event in NF-kappaB activation [corrected]. Nat. Cell Biol..

[56] Kanayama A., Seth R.B., Sun L., Ea C.-K., Hong M., Shaito A., Chiu Y.-H., Deng L., Chen Z.J. (2004). TAB2 and TAB3 activate the NF-kappaB pathway through binding to polyubiquitin chains. Mol. Cell.

[57] Iha H., Peloponese J.-M., Verstrepen L., Zapart G., Ikeda F., Smith C.D., Starost M.F., Yedavalli V., Heyninck K., Dikic I. (2008). Inflammatory cardiac valvulitis in TAX1BP1-deficient mice through selective NF-κB activation. EMBO J..

[58] Suryo Rahmanto A., Blum C.J., Scalera C., Heidelberger J.B., Mesitov M., Horn-Ghetko D., Gräf J.F., Mikicic I., Hobrecht R., Orekhova A. (2023). K6-linked ubiquitylation marks formaldehyde-induced RNA-protein crosslinks for resolution. Mol. Cell.

[59] Zhao S., Cordes J., Caban K.M., Götz M.J., Mackens-Kiani T., Veltri A.J., Sinha N.K., Weickert P., Kaya S., Hewitt G. (2023). RNF14-dependent atypical ubiquitylation promotes translation-coupled resolution of RNA-protein crosslinks. Mol. Cell.

[60] Tracz M., Bialek W. (2021). Beyond K48 and K63: Non-canonical protein ubiquitination. Cell Mol. Biol. Lett..

[61] Meyer H.-J., Rape M. (2014). Enhanced protein degradation by branched ubiquitin chains. Cell.

[62] Yau R.G., Doerner K., Castellanos E.R., Haakonsen D.L., Werner A., Wang N., Yang X.W., Martinez-Martin N., Matsumoto M.L., Dixit V.M. (2017). Assembly and Function of Heterotypic Ubiquitin Chains in Cell-Cycle and Protein Quality Control. Cell.

[63] Lamark T., Johansen T. (2012). Aggrephagy: Selective disposal of protein aggregates by macroautophagy. Int. J. Cell Biol..

[64] Bauer B., Martens S., Ferrari L. (2023). Aggrephagy at a glance. J. Cell Sci..

[65] Zhang Z., Klionsky D.J. (2022). CCT2, a newly identified aggrephagy receptor in mammals, specifically mediates the autophagic clearance of solid protein aggregates. Autophagy.

[66] Ma X., Lu C., Chen Y., Li S., Ma N., Tao X., Li Y., Wang J., Zhou M., Yan Y.-B. (2022). CCT2 is an aggrephagy receptor for clearance of solid protein aggregates. Cell.

[67] Sun D., Wu R., Zheng J., Li P., Yu L. (2018). Polyubiquitin chain-induced p62 phase separation drives autophagic cargo segregation. Cell Res..

[68] Zaffagnini G., Savova A., Danieli A., Romanov J., Tremel S., Ebner M., Peterbauer T., Sztacho M., Trapannone R., Tarafder A.K. (2018). p62 filaments capture and present ubiquitinated cargos for autophagy. EMBO J..

[69] Danieli A., Martens S. (2018). p62-mediated phase separation at the intersection of the ubiquitin-proteasome system and autophagy. J. Cell Sci..

[70] Turco E., Savova A., Gere F., Ferrari L., Romanov J., Schuschnig M., Martens S. (2021). Reconstitution defines the roles of p62, NBR1 and TAX1BP1 in ubiquitin condensate formation and autophagy initiation. Nat. Commun..

[71] Ferrari L., Bauer B., Qiu Y., Schuschnig M., Klotz S., Anrather D., Juretschke T., Beli P., Gelpi E., Martens S. (2024). Tau fibrils evade autophagy by excessive p62 coating and TAX1BP1 exclusion. Sci. Adv..

[72] Wegmann S., Eftekharzadeh B., Tepper K., Zoltowska K.M., Bennett R.E., Dujardin S., Laskowski P.R., MacKenzie D., Kamath T., Commins C. (2018). Tau protein liquid-liquid phase separation can initiate tau aggregation. EMBO J..

[73] Sarraf S.A., Shah H.V., Kanfer G., Pickrell A.M., Holtzclaw L.A., Ward M.E., Youle R.J. (2020). Loss of TAX1BP1-Directed Autophagy Results in Protein Aggregate Accumulation in the Brain. Mol. Cell.

[74] Saha I., Yuste-Checa P., Da Silva Padilha M., Guo Q., Körner R., Holthusen H., Trinkaus V.A., Dudanova I., Fernández-Busnadiego R., Baumeister W. (2023). The AAA+ chaperone VCP disaggregates Tau fibrils and generates aggregate seeds in a cellular system. Nat. Commun..

[75] Giong H.-K., Hyeon S.J., Lee J.-G., Cho H.-J., Park U., Stein T.D., Lee J., Yu K., Ryu H., Lee J.-S. (2024). Tau accumulation is cleared by the induced expression of VCP via autophagy. Acta Neuropathol..

[76] Zwang T.J., Woost B., Bailey J., Hoglund Z., Richardson D.S., Bennett R.E., Hyman B.T. (2023). Spatial characterization of tangle-bearing neurons and ghost tangles in the human inferior temporal gyrus with three-dimensional imaging. Brain Commun..

[77] de Calignon A., Spires-Jones T.L., Pitstick R., Carlson G.A., Hyman B.T. (2009). Tangle-bearing neurons survive despite disruption of membrane integrity in a mouse model of tauopathy. J. Neuropathol. Exp. Neurol..

[78] Du S., Wang Y., Chen B., Xie S., Chan K.Y., Hay D.C., Chew T.G. (2024). Clearance of protein aggregates during cell division. bioRxiv.

[79] Bhaskar K., Maphis N., Xu G., Varvel N.H., Kokiko-Cochran O.N., Weick J.P., Staugaitis S.M., Cardona A., Ransohoff R.M., Herrup K. (2014). Microglial derived tumor necrosis factor-α drives Alzheimer’s disease-related neuronal cell cycle events. Neurobiol. Dis..

[80] Ishikawa S., Ishikawa F. (2020). Proteostasis failure and cellular senescence in long-term cultured postmitotic rat neurons. Aging Cell.

[81] Musi N., Valentine J.M., Sickora K.R., Baeuerle E., Thompson C.S., Shen Q., Orr M.E. (2018). Tau protein aggregation is associated with cellular senescence in the brain. Aging Cell.

[82] Shay J.W., Wright W.E. (2000). Hayflick, his limit, and cellular ageing. Nat. Rev. Mol. Cell Biol..

[83] Chou S.-M., Yen Y.-H., Yuan F., Zhang S.-C., Chong C.-M. (2023). Neuronal Senescence in the Aged Brain. Aging Dis..

[84] Herdy J.R., Mertens J., Gage F.H. (2024). Neuronal senescence may drive brain aging. Science.

[85] Sah E., Krishnamurthy S., Ahmidouch M.Y., Gillispie G.J., Milligan C., Orr M.E. (2021). The Cellular Senescence Stress Response in Post-Mitotic Brain Cells: Cell Survival at the Expense of Tissue Degeneration. Life.

[86] Di Micco R., Krizhanovsky V., Baker D., d’Adda di Fagagna F. (2021). Cellular senescence in ageing: From mechanisms to therapeutic opportunities. Nat. Rev. Mol. Cell Biol..

[87] Ashraf H.M., Fernandez B., Spencer S.L. (2023). The intensities of canonical senescence biomarkers integrate the duration of cell-cycle withdrawal. Nat. Commun..

[88] Zhang L., Pitcher L.E., Yousefzadeh M.J., Niedernhofer L.J., Robbins P.D., Zhu Y. (2022). Cellular senescence: A key therapeutic target in aging and diseases. J. Clin. Investig..

[89] Muthamil S., Kim H.Y., Jang H.J., Lyu J.H., Shin U.C., Go Y., Park S.H., Lee H.G., Park J.H. (2024). Biomarkers of Cellular Senescence and Aging: Current State-of-the-Art, Challenges and Future Perspectives. Adv. Biol..

[90] Admasu T.D., Rae M., Stolzing A. (2021). Dissecting primary and secondary senescence to enable new senotherapeutic strategies. Ageing Res. Rev..

[91] Mayford M., Siegelbaum S.A., Kandel E.R. (2012). Synapses and memory storage. Cold Spring Harb. Perspect. Biol..

[92] Fields R.D. (2022). The Enigma of Working Memory: Changing Views. Neurosci. Rev. J. Bringing Neurobiol. Neurol. Psychiatry.

[93] Sanhedrai H., Havlin S., Dvir H. (2024). Mechanistic description of spontaneous loss of memory persistent activity based on neuronal synaptic strength. Heliyon.

[94] Häusser M., Raman I.M., Otis T., Smith S.L., Nelson A., Du Lac S., Loewenstein Y., Mahon S., Pennartz C., Cohen I. (2004). The beat goes on: Spontaneous firing in mammalian neuronal microcircuits. J. Neurosci. Off. J. Soc. Neurosci..

[95] Kirova A.-M., Bays R.B., Lagalwar S. (2015). Working Memory and Executive Function Decline across Normal Aging, Mild Cognitive Impairment, and Alzheimer’s Disease. BioMed Res. Int..

[96] Stopford C.L., Thompson J.C., Neary D., Richardson A.M.T., Snowden J.S. (2012). Working memory, attention, and executive function in Alzheimer’s disease and frontotemporal dementia. Cortex.

[97] Herdy J.R., Traxler L., Agarwal R.K., Karbacher L., Schlachetzki J.C.M., Boehnke L., Zangwill D., Galasko D., Glass C.K., Mertens J. (2022). Increased post-mitotic senescence in aged human neurons is a pathological feature of Alzheimer’s disease. Cell Stem Cell.

[98] Dehkordi S.K., Walker J., Sah E., Bennett E., Atrian F., Frost B., Woost B., Bennett R.E., Orr T.C., Zhou Y. (2021). Profiling senescent cells in human brains reveals neurons with CDKN2D/p19 and tau neuropathology. Nat. Aging.

[99] Ota Y., Zanetti A.T., Hallock R.M. (2013). The role of astrocytes in the regulation of synaptic plasticity and memory formation. Neural Plast..

[100] Han X., Zhang T., Liu H., Mi Y., Gou X. (2020). Astrocyte Senescence and Alzheimer’s Disease: A Review. Front. Aging Neurosci..

[101] Esposito Z., Belli L., Toniolo S., Sancesario G., Bianconi C., Martorana A. (2013). Amyloid β, Glutamate, Excitotoxicity in Alzheimer’s Disease: Are We on the Right Track?. CNS Neurosci. Ther..

[102] Kim J., Yoo I.D., Lim J., Moon J.-S. (2024). Pathological phenotypes of astrocytes in Alzheimer’s disease. Exp. Mol. Med..

[103] Wang R., Reddy P.H. (2017). Role of Glutamate and NMDA Receptors in Alzheimer’s Disease. J. Alzheimers Dis..

[104] Bhat R., Crowe E.P., Bitto A., Moh M., Katsetos C.D., Garcia F.U., Johnson F.B., Trojanowski J.Q., Sell C., Torres C. (2012). Astrocyte Senescence as a Component of Alzheimer’s Disease. PLoS ONE.

[105] Limbad C., Oron T.R., Alimirah F., Davalos A.R., Tracy T.E., Gan L., Desprez P.-Y., Campisi J. (2020). Astrocyte senescence promotes glutamate toxicity in cortical neurons. PLoS ONE.

[106] Bitto A., Sell C., Crowe E., Lorenzini A., Malaguti M., Hrelia S., Torres C. (2010). Stress-induced senescence in human and rodent astrocytes. Exp. Cell Res..

[107] Gaikwad S., Puangmalai N., Bittar A., Montalbano M., Garcia S., McAllen S., Bhatt N., Sonawane M., Sengupta U., Kayed R. (2021). Tau oligomer induced HMGB1 release contributes to cellular senescence and neuropathology linked to Alzheimer’s disease and frontotemporal dementia. Cell Rep..

[108] Simons M., Nave K.-A. (2015). Oligodendrocytes: Myelination and Axonal Support. Cold Spring Harb. Perspect. Biol..

[109] Kuhn S., Gritti L., Crooks D., Dombrowski Y. (2019). Oligodendrocytes in Development, Myelin Generation and Beyond. Cells.

[110] Garton T., Gadani S.P., Gill A.J., Calabresi P.A. (2024). Neurodegeneration and demyelination in multiple sclerosis. Neuron.

[111] Costa V.G.C., Araújo S.E.-S., Alves-Leon S.V., Gomes F.C.A. (2023). Central nervous system demyelinating diseases: Glial cells at the hub of pathology. Front. Immunol..

[112] LaCroix M.S., Mirbaha H., Shang P., Zandee S., Foong C., Prat A., White C.L., Stuve O., Diamond M.I. (2022). Tau seeding in cases of multiple sclerosis. Acta Neuropathol. Commun..

[113] Zhou T., Ahmad T.K., Gozda K., Truong J., Kong J., Namaka M. (2017). Implications of white matter damage in amyotrophic lateral sclerosis (Review). Mol. Med. Rep..

[114] Huang Z., Jordan J.D., Zhang Q. (2024). Myelin Pathology in Alzheimer’s Disease: Potential Therapeutic Opportunities. Aging Dis..

[115] Torii T., Miyamoto Y., Nakata R., Higashi Y., Shinmyo Y., Kawasaki H., Miyasaka T., Misonou H. (2023). Identification of Tau protein as a novel marker for maturation and pathological changes of oligodendrocytes. Glia.

[116] Harada A., Oguchi K., Okabe S., Kuno J., Terada S., Ohshima T., Sato-Yoshitake R., Takei Y., Noda T., Hirokawa N. (1994). Altered microtubule organization in small-calibre axons of mice lacking tau protein. Nature.

[117] Seiberlich V., Bauer N.G., Schwarz L., Ffrench-Constant C., Goldbaum O., Richter-Landsberg C. (2015). Downregulation of the microtubule associated protein T au impairs process outgrowth and myelin basic protein m RNA transport in oligodendrocytes. Glia.

[118] Torii T. (2024). Abnormal expression of Tau in damaged oligodendrocytes of HLD1 mice. Neural Regen. Res..

[119] De Rossi P., Buggia-Prévot V., Clayton B.L.L., Vasquez J.B., van Sanford C., Andrew R.J., Lesnick R., Botté A., Deyts C., Salem S. (2016). Predominant expression of Alzheimer’s disease-associated BIN1 in mature oligodendrocytes and localization to white matter tracts. Mol. Neurodegener..

[120] Sottejeau Y., Bretteville A., Cantrelle F.-X., Malmanche N., Demiaute F., Mendes T., Delay C., Alves Dos Alves H., Flaig A., Davies P. (2015). Tau phosphorylation regulates the interaction between BIN1’s SH3 domain and Tau’s proline-rich domain. Acta Neuropathol. Commun..

[121] Gautier H.O.B., Evans K.A., Volbracht K., James R., Sitnikov S., Lundgaard I., James F., Lao-Peregrin C., Reynolds R., Franklin R.J.M. (2015). Neuronal activity regulates remyelination via glutamate signalling to oligodendrocyte progenitors. Nat. Commun..

[122] Viney T.J., Sarkany B., Ozdemir A.T., Hartwich K., Schweimer J., Bannerman D., Somogyi P. (2022). Spread of pathological human Tau from neurons to oligodendrocytes and loss of high-firing pyramidal neurons in aging mice. Cell Rep..

[123] Narasimhan S., Changolkar L., Riddle D.M., Kats A., Stieber A., Weitzman S.A., Zhang B., Li Z., Roberson E.D., Trojanowski J.Q. (2020). Human tau pathology transmits glial tau aggregates in the absence of neuronal tau. J. Exp. Med..

[124] Sasmita A.O., Depp C., Nazarenko T., Sun T., Siems S.B., Ong E.C., Nkeh Y.B., Böhler C., Yu X., Bues B. (2024). Oligodendrocytes produce amyloid-β and contribute to plaque formation alongside neurons in Alzheimer’s disease model mice. Nat. Neurosci..

[125] Zhang P., Kishimoto Y., Grammatikakis I., Gottimukkala K., Cutler R.G., Zhang S., Abdelmohsen K., Bohr V.A., Misra Sen J., Gorospe M. (2019). Senolytic therapy alleviates Aβ-associated oligodendrocyte progenitor cell senescence and cognitive deficits in an Alzheimer’s disease model. Nat. Neurosci..

[126] Rouillard M.E., Hu J., Sutter P.A., Kim H.W., Huang J.K., Crocker S.J. (2022). The Cellular Senescence Factor Extracellular HMGB1 Directly Inhibits Oligodendrocyte Progenitor Cell Differentiation and Impairs CNS Remyelination. Front. Cell. Neurosci..

[127] Roy E.R., Chiu G., Li S., Propson N.E., Kanchi R., Wang B., Coarfa C., Zheng H., Cao W. (2022). Concerted type I interferon signaling in microglia and neural cells promotes memory impairment associated with amyloid β plaques. Immunity.

[128] Jin M., Xu R., Wang L., Alam M.M., Ma Z., Zhu S., Martini A.C., Jadali A., Bernabucci M., Xie P. (2022). Type-I-interferon signaling drives microglial dysfunction and senescence in human iPSC models of Down syndrome and Alzheimer’s disease. Cell Stem Cell.

[129] Lana-Elola E., Watson-Scales S.D., Fisher E.M.C., Tybulewicz V.L.J. (2011). Down syndrome: Searching for the genetic culprits. Dis. Model. Mech..

[130] Hong S., Beja-Glasser V.F., Nfonoyim B.M., Frouin A., Li S., Ramakrishnan S., Merry K.M., Shi Q., Rosenthal A., Barres B.A. (2016). Complement and microglia mediate early synapse loss in Alzheimer mouse models. Science.

[131] Peng Q., Malhotra S., Torchia J.A., Kerr W.G., Coggeshall K.M., Humphrey M.B. (2010). TREM2- and DAP12-Dependent Activation of PI3K Requires DAP10 and Is Inhibited by SHIP1. Sci. Signal..

[132] Wißfeld J., Mathews M., Mossad O., Picardi P., Cinti A., Redaelli L., Pradier L., Brüstle O., Neumann H. (2021). Reporter cell assay for human CD33 validated by specific antibodies and human iPSC-derived microglia. Sci. Rep..

[133] Mócsai A., Abram C.L., Jakus Z., Hu Y., Lanier L.L., Lowell C.A. (2006). Integrin signaling in neutrophils and macrophages uses adaptors containing immunoreceptor tyrosine-based activation motifs. Nat. Immunol..

[134] Gaikwad S., Larionov S., Wang Y., Dannenberg H., Matozaki T., Monsonego A., Thal D.R., Neumann H. (2009). Signal regulatory protein-beta1: A microglial modulator of phagocytosis in Alzheimer’s disease. Am. J. Pathol..

[135] Haure-Mirande J.-V., Audrain M., Fanutza T., Kim S.H., Klein W.L., Glabe C., Readhead B., Dudley J.T., Blitzer R.D., Wang M. (2017). Deficiency of TYROBP, an adapter protein for TREM2 and CR3 receptors, is neuroprotective in a mouse model of early Alzheimer’s pathology. Acta Neuropathol..

[136] Audrain M., Haure-Mirande J.V., Wang M., Kim S.H., Fanutza T., Chakrabarty P., Fraser P., St George-Hyslop P.H., Golde T.E., Blitzer R.D. (2019). Integrative approach to sporadic Alzheimer’s disease: Deficiency of TYROBP in a tauopathy mouse model reduces C1q and normalizes clinical phenotype while increasing spread and state of phosphorylation of tau. Mol. Psychiatry.

[137] Haure-Mirande J.V., Wang M., Audrain M., Fanutza T., Kim S.H., Heja S., Readhead B., Dudley J.T., Blitzer R.D., Schadt E.E. (2019). Integrative approach to sporadic Alzheimer’s disease: Deficiency of TYROBP in cerebral Aβ amyloidosis mouse normalizes clinical phenotype and complement subnetwork molecular pathology without reducing Aβ burden. Mol. Psychiatry.

[138] Yuan P., Condello C., Keene C.D., Wang Y., Bird T.D., Paul S.M., Luo W., Colonna M., Baddeley D., Grutzendler J. (2016). TREM2 Haplodeficiency in Mice and Humans Impairs the Microglia Barrier Function Leading to Decreased Amyloid Compaction and Severe Axonal Dystrophy. Neuron.

[139] Rachmian N., Medina S., Cherqui U., Akiva H., Deitch D., Edilbi D., Croese T., Salame T.M., Ramos J.M.P., Cahalon L. (2024). Identification of senescent, TREM2-expressing microglia in aging and Alzheimer’s disease model mouse brain. Nat. Neurosci..

[140] Koenigsknecht J., Landreth G. (2004). Microglial phagocytosis of fibrillar beta-amyloid through a beta1 integrin-dependent mechanism. J. Neurosci..

[141] Mandrekar S., Jiang Q., Lee C.Y.D., Koenigsknecht-Talboo J., Holtzman D.M., Landreth G.E. (2009). Microglia mediate the clearance of soluble Abeta through fluid phase macropinocytosis. J. Neurosci..

[142] Condello C., Yuan P., Schain A., Grutzendler J. (2015). Microglia constitute a barrier that prevents neurotoxic protofibrillar Aβ42 hotspots around plaques. Nat. Commun..

[143] Yang C.N., Shiao Y.J., Shie F.S., Guo B.S., Chen P.H., Cho C.Y., Chen Y.J., Huang F.L., Tsay H.J. (2011). Mechanism mediating oligomeric Aβ clearance by naïve primary microglia. Neurobiol. Dis..

[144] Banati R.B., Rothe G., Valet G., Kreutzberg G.W. (1993). Detection of lysosomal cysteine proteinases in microglia: Flow cytometric measurement and histochemical localization of cathepsin B and L. Glia.

[145] Hu Y., Fryatt G.L., Ghorbani M., Obst J., Menassa D.A., Martin-Estebane M., Muntslag T.A.O., Olmos-Alonso A., Guerrero-Carrasco M., Thomas D. (2021). Replicative senescence dictates the emergence of disease-associated microglia and contributes to Aβ pathology. Cell Rep..

[146] Paolicelli R.C., Sierra A., Stevens B., Tremblay M.E., Aguzzi A., Ajami B., Amit I., Audinat E., Bechmann I., Bennett M. (2022). Microglia states and nomenclature: A field at its crossroads. Neuron.

[147] Olmos-Alonso A., Schetters S.T., Sri S., Askew K., Mancuso R., Vargas-Caballero M., Holscher C., Perry V.H., Gomez-Nicola D. (2016). Pharmacological targeting of CSF1R inhibits microglial proliferation and prevents the progression of Alzheimer’s-like pathology. Brain.

[148] Easley-Neal C., Foreman O., Sharma N., Zarrin A.A., Weimer R.M. (2019). CSF1R Ligands IL-34 and CSF1 Are Differentially Required for Microglia Development and Maintenance in White and Gray Matter Brain Regions. Front. Immunol..

[149] Füger P., Hefendehl J.K., Veeraraghavalu K., Wendeln A.-C., Schlosser C., Obermüller U., Wegenast-Braun B.M., Neher J.J., Martus P., Kohsaka S. (2017). Microglia turnover with aging and in an Alzheimer’s model via long-term in vivo single-cell imaging. Nat. Neurosci..

[150] Deczkowska A., Keren-Shaul H., Weiner A., Colonna M., Schwartz M., Amit I. (2018). Disease-Associated Microglia: A Universal Immune Sensor of Neurodegeneration. Cell.

[151] Choi I., Wang M., Yoo S., Xu P., Seegobin S.P., Li X., Han X., Wang Q., Peng J., Zhang B. (2023). Autophagy enables microglia to engage amyloid plaques and prevents microglial senescence. Nat. Cell Biol..

[152] Amin S., Liu B., Gan L. (2023). Autophagy prevents microglial senescence. Nat. Cell Biol..

[153] Karabag D., Scheiblich H., Griep A., Santarelli F., Schwartz S., Heneka M.T., Ising C. (2023). Characterizing microglial senescence: Tau as a key player. J. Neurochem..

[154] Matsudaira T., Nakano S., Konishi Y., Kawamoto S., Uemura K., Kondo T., Sakurai K., Ozawa T., Hikida T., Komine O. (2023). Cellular senescence in white matter microglia is induced during ageing in mice and exacerbates the neuroinflammatory phenotype. Commun. Biol..

[155] Baker D.J., Wijshake T., Tchkonia T., LeBrasseur N.K., Childs B.G., van de Sluis B., Kirkland J.L., van Deursen J.M. (2011). Clearance of p16Ink4a-positive senescent cells delays ageing-associated disorders. Nature.

[156] Bussian T.J., Aziz A., Meyer C.F., Swenson B.L., van Deursen J.M., Baker D.J. (2018). Clearance of senescent glial cells prevents tau-dependent pathology and cognitive decline. Nature.

[157] Ogrodnik M., Evans S.A., Fielder E., Victorelli S., Kruger P., Salmonowicz H., Weigand B.M., Patel A.D., Pirtskhalava T., Inman C.L. (2021). Whole-body senescent cell clearance alleviates age-related brain inflammation and cognitive impairment in mice. Aging Cell.

[158] Wu D., Sun J.K.-L., Chow K.H.-M. (2024). Neuronal cell cycle reentry events in the aging brain are more prevalent in neurodegeneration and lead to cellular senescence. PLoS Biol..

[159] Sabath N., Levy-Adam F., Younis A., Rozales K., Meller A., Hadar S., Soueid-Baumgarten S., Shalgi R. (2020). Cellular proteostasis decline in human senescence. Proc. Natl. Acad. Sci. USA.

[160] Husom A.D., Peters E.A., Kolling E.A., Fugere N.A., Thompson L.V., Ferrington D.A. (2004). Altered proteasome function and subunit composition in aged muscle. Arch. Biochem. Biophys..

[161] Strucksberg K.-H., Tangavelou K., Schröder R., Clemen C.S. (2010). Proteasomal activity in skeletal muscle: A matter of assay design, muscle type, and age. Anal. Biochem..

[162] Nago N., Murata S., Tanaka K., Tanahashi N. (2024). Changes in brain proteasome dynamics associated with aging. Genes Cells.

[163] Keck S., Nitsch R., Grune T., Ullrich O. (2003). Proteasome inhibition by paired helical filament-tau in brains of patients with Alzheimer’s disease. J. Neurochem..

[164] Chondrogianni N., Stratford F.L.L., Trougakos I.P., Friguet B., Rivett A.J., Gonos E.S. (2003). Central role of the proteasome in senescence and survival of human fibroblasts: Induction of a senescence-like phenotype upon its inhibition and resistance to stress upon its activation. J. Biol. Chem..

[165] Chondrogianni N., Gonos E.S. (2004). Proteasome inhibition induces a senescence-like phenotype in primary human fibroblasts cultures. Biogerontology.

[166] Torres C., Lewis L., Cristofalo V.J. (2006). Proteasome inhibitors shorten replicative life span and induce a senescent-like phenotype of human fibroblasts. J. Cell. Physiol..

[167] Takenaka Y., Inoue I., Nakano T., Ikeda M., Kakinuma Y. (2022). Prolonged disturbance of proteostasis induces cellular senescence via temporal mitochondrial dysfunction and subsequent mitochondrial accumulation in human fibroblasts. FEBS J..

[168] Yasuda S., Tsuchiya H., Kaiho A., Guo Q., Ikeuchi K., Endo A., Arai N., Ohtake F., Murata S., Inada T. (2020). Stress- and ubiquitylation-dependent phase separation of the proteasome. Nature.

[169] Iriki T., Iio H., Yasuda S., Masuta S., Kato M., Kosako H., Hirayama S., Endo A., Ohtake F., Kamiya M. (2023). Senescent cells form nuclear foci that contain the 26S proteasome. Cell Rep..

[170] Heckenbach I., Mkrtchyan G.V., Ezra M.B., Bakula D., Madsen J.S., Nielsen M.H., Oró D., Osborne B., Covarrubias A.J., Idda M.L. (2022). Nuclear morphology is a deep learning biomarker of cellular senescence. Nat. Aging.

[171] Freund A., Laberge R.-M., Demaria M., Campisi J. (2012). Lamin B1 loss is a senescence-associated biomarker. Mol. Biol. Cell.

[172] Slobodnyuk K., Radic N., Ivanova S., Llado A., Trempolec N., Zorzano A., Nebreda A.R. (2019). Autophagy-induced senescence is regulated by p38α signaling. Cell Death Dis..

[173] Dou Z., Xu C., Donahue G., Shimi T., Pan J.-A., Zhu J., Ivanov A., Capell B.C., Drake A.M., Shah P.P. (2015). Autophagy mediates degradation of nuclear lamina. Nature.

[174] Kang H.T., Lee K.B., Kim S.Y., Choi H.R., Park S.C. (2011). Autophagy impairment induces premature senescence in primary human fibroblasts. PLoS ONE.

[175] Frost B., Bardai F.H., Feany M.B. (2016). Lamin Dysfunction Mediates Neurodegeneration in Tauopathies. Curr. Biol..

[176] Li Y., Jiang X., Zhang Y., Gao Z., Liu Y., Hu J., Hu X., Li L., Shi J., Gao N. (2019). Nuclear accumulation of UBC9 contributes to SUMOylation of lamin A/C and nucleophagy in response to DNA damage. J. Exp. Clin. Cancer Res..

[177] En A., Takauji Y., Miki K., Ayusawa D., Fujii M. (2020). Lamin B receptor plays a key role in cellular senescence induced by inhibition of the proteasome. FEBS Open Bio.

[178] Lukášová E., Kovařík A., Bačíková A., Falk M., Kozubek S. (2017). Loss of lamin B receptor is necessary to induce cellular senescence. Biochem. J..

[179] Arai R., En A., Takauji Y., Maki K., Miki K., Fujii M., Ayusawa D. (2019). Lamin B receptor (LBR) is involved in the induction of cellular senescence in human cells. Mech. Ageing Dev..

[180] Arai R., En A., Ukekawa R., Miki K., Fujii M., Ayusawa D. (2016). Aberrant localization of lamin B receptor (LBR) in cellular senescence in human cells. Biochem. Biophys. Res. Commun..

[181] De Sandre-Giovannoli A., Bernard R., Cau P., Navarro C., Amiel J., Boccaccio I., Lyonnet S., Stewart C.L., Munnich A., Le Merrer M. (2003). Lamin a truncation in Hutchinson-Gilford progeria. Science.

[182] Eriksson M., Brown W.T., Gordon L.B., Glynn M.W., Singer J., Scott L., Erdos M.R., Robbins C.M., Moses T.Y., Berglund P. (2003). Recurrent de novo point mutations in lamin A cause Hutchinson-Gilford progeria syndrome. Nature.

[183] Anderson K.A., Madsen A.S., Olsen C.A., Hirschey M.D. (2017). Metabolic control by sirtuins and other enzymes that sense NAD+, NADH, or their ratio. Biochim. Biophys. Acta Bioenerg..

[184] Jing H., Lin H. (2015). Sirtuins in epigenetic regulation. Chem. Rev..

[185] Yeung F., Hoberg J.E., Ramsey C.S., Keller M.D., Jones D.R., Frye R.A., Mayo M.W. (2004). Modulation of NF-kappaB-dependent transcription and cell survival by the SIRT1 deacetylase. EMBO J..

[186] Xu C., Wang L., Fozouni P., Evjen G., Chandra V., Jiang J., Lu C., Nicastri M., Bretz C., Winkler J.D. (2020). SIRT1 is downregulated by autophagy in senescence and ageing. Nat. Cell Biol..

[187] Narita M., Young A.R.J., Arakawa S., Samarajiwa S.A., Nakashima T., Yoshida S., Hong S., Berry L.S., Reichelt S., Ferreira M. (2011). Spatial Coupling of mTOR and Autophagy Augments Secretory Phenotypes. Science.

[188] Tan J.X., Finkel T. (2023). Lysosomes in senescence and aging. EMBO Rep..

[189] Curnock R., Yalci K., Palmfeldt J., Jäättelä M., Liu B., Carroll B. (2023). TFEB-dependent lysosome biogenesis is required for senescence. EMBO J..

[190] Nishida Y., Arakawa S., Fujitani K., Yamaguchi H., Mizuta T., Kanaseki T., Komatsu M., Otsu K., Tsujimoto Y., Shimizu S. (2009). Discovery of Atg5/Atg7-independent alternative macroautophagy. Nature.

[191] Yamaguchi H., Arakawa S., Kanaseki T., Miyatsuka T., Fujitani Y., Watada H., Tsujimoto Y., Shimizu S. (2016). Golgi membrane-associated degradation pathway in yeast and mammals. EMBO J..

[192] Baron O., Boudi A., Dias C., Schilling M., Nölle A., Vizcay-Barrena G., Rattray I., Jungbluth H., Scheper W., Fleck R.A. (2017). Stall in Canonical Autophagy-Lysosome Pathways Prompts Nucleophagy-Based Nuclear Breakdown in Neurodegeneration. Curr. Biol..

[193] Van Acker Z.P., Bretou M., Annaert W. (2019). Endo-lysosomal dysregulations and late-onset Alzheimer’s disease: Impact of genetic risk factors. Mol. Neurodegener..

[194] Karch C.M., Goate A.M. (2015). Alzheimer’s Disease Risk Genes and Mechanisms of Disease Pathogenesis. Biol. Psychiatry.

[195] Langerscheidt F., Wied T., Al Kabbani M.A., van Eimeren T., Wunderlich G., Zempel H. (2024). Genetic forms of tauopathies: Inherited causes and implications of Alzheimer’s disease-like TAU pathology in primary and secondary tauopathies. J. Neurol..

[196] Clayton E.L., Mizielinska S., Edgar J.R., Nielsen T.T., Marshall S., Norona F.E., Robbins M., Damirji H., Holm I.E., The FReJA Consortium (2015). Frontotemporal dementia caused by CHMP2B mutation is characterised by neuronal lysosomal storage pathology. Acta Neuropathol..

[197] Clayton E.L., Milioto C., Muralidharan B., Norona F.E., Edgar J.R., Soriano A., Jafar-Nejad P., Rigo F., Collinge J., Isaacs A.M. (2018). Frontotemporal dementia causative CHMP2B impairs neuronal endolysosomal traffic-rescue by *TMEM106B* knockdown. Brain.

[198] Midani-Kurçak J.S., Dinekov M., Puladi B., Arzberger T., Köhler C. (2019). Effect of tau-pathology on charged multivesicular body protein 2b (CHMP2B). Brain Res..

[199] Zhang Y., Schmid B., Nikolaisen N.K., Rasmussen M.A., Aldana B.I., Agger M., Calloe K., Stummann T.C., Larsen H.M., Nielsen T.T. (2017). Patient iPSC-Derived Neurons for Disease Modeling of Frontotemporal Dementia with Mutation in CHMP2B. Stem Cell Rep..

[200] Lin B.C., Higgins N.R., Phung T.H., Monteiro M.J. (2022). UBQLN proteins in health and disease with a focus on UBQLN2 in ALS/FTD. FEBS J..

[201] Gerson J.E., Sandoval-Pistorius S., Welday J.P., Rodriguez A., Gregory J.D., Liggans N., Schache K., Li X., Trzeciakiewicz H., Barmada S. (2022). Disrupting the Balance of Protein Quality Control Protein UBQLN2 Accelerates Tau Proteinopathy. J. Neurosci..

[202] Xia Y. (2022). Role of Ubiquilin-2 in Proteostasis and Tau Aggregation in Tauopathies. J. Neurosci..

[203] Thumbadoo K.M., Dieriks B.V., Murray H.C., Swanson M.E.V., Yoo J.H., Mehrabi N.F., Turner C., Dragunow M., Faull R.L.M., Curtis M.A. (2024). Hippocampal aggregation signatures of pathogenic UBQLN2 in amyotrophic lateral sclerosis and frontotemporal dementia. Brain.

[204] Darwich N.F., Phan J.M., Kim B., Suh E., Papatriantafyllou J.D., Changolkar L., Nguyen A.T., O’rourke C.M., He Z., Porta S. (2020). Autosomal dominant VCP hypomorph mutation impairs disaggregation of PHF-tau. Science.

[205] Le Ber I., Camuzat A., Guerreiro R., Bouya-Ahmed K., Bras J., Nicolas G., Gabelle A., Didic M., De Septenville A., Millecamps S. (2013). SQSTM1 Mutations in French Patients With Frontotemporal Dementia or Frontotemporal Dementia With Amyotrophic Lateral Sclerosis. JAMA Neurol..

[206] Deng Z., Lim J., Wang Q., Purtell K., Wu S., Palomo G.M., Tan H., Manfredi G., Zhao Y., Peng J. (2020). ALS-FTLD-linked mutations of SQSTM1/p62 disrupt selective autophagy and NFE2L2/NRF2 anti-oxidative stress pathway. Autophagy.

[207] Ono M., Komatsu M., Ji B., Takado Y., Shimojo M., Minamihisamatsu T., Warabi E., Yanagawa T., Matsumoto G., Aoki I. (2022). Central role for p62/SQSTM1 in the elimination of toxic tau species in a mouse model of tauopathy. Aging Cell.

[208] Xu Y., Zhang S., Zheng H. (2019). The cargo receptor SQSTM1 ameliorates neurofibrillary tangle pathology and spreading through selective targeting of pathological MAPT (microtubule associated protein tau). Autophagy.

[209] Herbst S., Lewis P.A., Morris H.R. (2022). The emerging role of LRRK2 in tauopathies. Clin. Sci..

[210] Madureira M., Connor-Robson N., Wade-Martins R. (2020). LRRK2: Autophagy and Lysosomal Activity. Front. Neurosci..

[211] Valentino R.R., Scotton W.J., Roemer S.F., Lashley T., Heckman M.G., Shoai M., Martinez-Carrasco A., Tamvaka N., Walton R.L., Baker M.C. (2024). MAPT H2 haplotype and risk of Pick’s disease in the Pick’s disease International Consortium: A genetic association study. Lancet Neurol..

[212] Caffrey T.M., Wade-Martins R. (2007). Functional MAPT haplotypes: Bridging the gap between genotype and neuropathology. Neurobiol. Dis..

[213] Sánchez-Juan P., Moreno S., de Rojas I., Hernández I., Valero S., Alegret M., Montrreal L., González P.G., Lage C., López-García S. (2019). The MAPT H1 Haplotype Is a Risk Factor for Alzheimer’s Disease in APOE ε4 Non-carriers. Front. Aging Neurosci..

[214] Bieniek K.F., Murray M.E., Rutherford N.J., Castanedes-Casey M., DeJesus-Hernandez M., Liesinger A.M., Baker M.C., Boylan K.B., Rademakers R., Dickson D.W. (2013). Tau pathology in frontotemporal lobar degeneration with C9ORF72 hexanucleotide repeat expansion. Acta Neuropathol..

[215] Tomé S.O., Tsaka G., Ronisz A., Ospitalieri S., Gawor K., Gomes L.A., Otto M., von Arnim C.A.F., Van Damme P., Bosch L.V.D. (2023). TDP-43 pathology is associated with increased tau burdens and seeding. Mol. Neurodegener..

[216] Chornenkyy Y., Fardo D.W., Nelson P.T. (2019). Tau and TDP-43 proteinopathies: Kindred pathologic cascades and genetic pleiotropy. Mod. Pathol..

[217] Gaikwad S., Senapati S., Haque A., Kayed R. (2024). Senescence, brain inflammation, and oligomeric tau drive cognitive decline in Alzheimer’s disease: Evidence from clinical and preclinical studies. Alzheimer’s Dement..

[218] Riessland M., Ximerakis M., Jarjour A.A., Zhang B., Orr M.E. (2024). Therapeutic targeting of senescent cells in the CNS. Nat. Rev. Drug Discov..

[219] Abisambra J.F., Jinwal U.K., Blair L.J., O’Leary J.C., Li Q., Brady S., Wang L., Guidi C.E., Zhang B., Nordhues B.A. (2013). Tau Accumulation Activates the Unfolded Protein Response by Impairing Endoplasmic Reticulum-Associated Degradation. J. Neurosci..

[220] Murray H.C., Dieriks B.V., Swanson M.E.V., Anekal P.V., Turner C., Faull R.L.M., Belluscio L., Koretsky A., Curtis M.A. (2020). The unfolded protein response is activated in the olfactory system in Alzheimer’s disease. Acta Neuropathol. Commun..

[221] van der Harg J.M., Nölle A., Zwart R., Boerema A.S., van Haastert E.S., Strijkstra A.M., Hoozemans J.J., Scheper W. (2014). The unfolded protein response mediates reversible tau phosphorylation induced by metabolic stress. Cell Death Dis..

[222] Ajoolabady A., Lindholm D., Ren J., Pratico D. (2022). ER stress and UPR in Alzheimer’s disease: Mechanisms, pathogenesis, treatments. Cell Death Dis..

[223] Pitera A.P., Asuni A.A., O’connor V., Deinhardt K. (2019). Pathogenic tau does not drive activation of the unfolded protein response. J. Biol. Chem..

